# Photoelectrocatalytic urea synthesis: Surface engineering of the constructions, mechanism insights, and technological challenges

**DOI:** 10.1016/j.isci.2026.116683

**Published:** 2026-07-30

**Authors:** Mohsen Padervand, Eric Lichtfouse, Shahnaz Ghasemi, Ali Abdollahi, Abdelkader Labidi, Aziz Habibi-Yangjeh, Elmuez A. Dawi, Michela Signoretto, Likun Pan, Chuanyi Wang

**Affiliations:** 1 Department of Chemistry, Faculty of Science, University of Maragheh, Maragheh, Iran; 2 School of Environmental Science and Engineering, Shaanxi University of Science and Technology, Xi’an 710021, China; 3 State Key Laboratory of Multiphase Flow in Power Engineering, Xi’an Jiaotong University, Xi’an, Shaanxi 710049, China; 4 Sharif Energy, Water and Environment Institute, Sharif University of Technology, Azadi Avenue, P.O. Box 11365-8639, Tehran, Iran; 5 Faculty of Technical and Engineering, Imam Khomeini International University, Qazvin, Iran; 6 Department of Chemistry, Faculty of Science, University of Mohaghegh Ardabili, Ardabil, Iran; 7 College of Humanities and Sciences, Department of Mathematics and Science, P.O. Box 346, Ajman, United Arab Emirates; 8 CATMAT Lab, Department of Molecular Sciences and Nanosystems Ca’ Foscari University Venice and INSTM Consortium, RU of Venice, Via Torino 155, 30172 Venezia Mestre, Italy; 9 Shanghai Key Laboratory of Magnetic Resonance, School of Physics , East China Normal University, Shanghai 200241, China

**Keywords:** Photoelectrocatalysis, Urea, Mechanism, Selectivity, Sustainability

## Abstract

Traditional urea production methods, primarily the Haber-Bosch and Bosch-Meiser processes, consume substantial amounts of energy and have significant environmental impacts, calling for more sustainable alternatives. Alternatively, photoelectrocatalytic (PEC) urea synthesis utilizes sunlight and electrochemistry to facilitate C–N coupling reactions under mild, environmentally friendly conditions. This review focuses on the recent progress in electrocatalytic, photocatalytic, and photoelectrocatalytic urea synthesis, with a focus on methodology, technological constraints and innovations. Key limitations include low Faradaic efficiencies, restricted catalytic selectivity and mechanistic understanding, and suboptimal charge-transfer processes. We discuss the potential of several photo-electroactive constructions, and quantification methodologies, highlighting a need for standardized testing protocols. Economic aspects of the technology are also discussed, with focus on the industrial feasibility of green urea production. Although PEC synthesis offers notable benefits over conventional methods, it still faces challenges, including catalyst durability, high energy consumption, and limited scalability. This review provides strategies to overcome these challenges and advance PEC urea synthesis toward real-world implementation in sustainable fertilizer manufacturing.

## Graphical abstract

**Figure:**
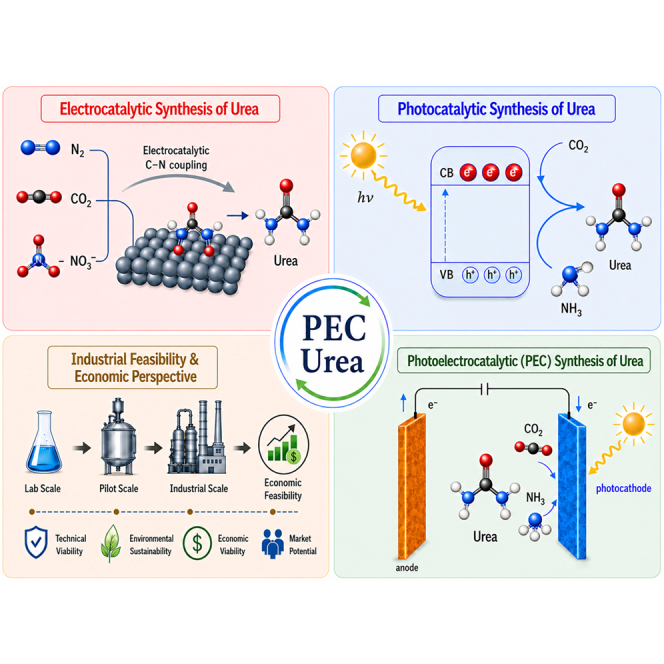

## Teaser

Catalysis; Theoretical photochemistry; Analytical Electrochemistry

## Introduction

Urea is classified as a significant component in the human food chain. According to reports, the global demand for urea is expected to reach 200 million metric tons by 2026. Urea, widely used as an organic nitrogen fertilizer, has a significant impact on the health-related quality of life for over 50% of the global population.^1^ Urea is a primary raw material for the fabrication of a wide range of fine chemicals used in medicine, agriculture, and various industrial sectors, including plastics, resins, and adhesives.^2^ Other fascinating applications of this compound include its utilization in sensor design for enzymatic reactions^3^ and fuel cell technologies.^4^^,^^5^ The industrial-scale production of urea relies on the direct reaction of ammonia (NH_3_) and carbon dioxide (CO_2_) under extreme conditions, including high temperatures and pressures, as well as specialized reactors.^6^ The NH_3_ used in urea production is primarily produced from N_2_ and H_2_ through various reactions, including the Bosch-Meiser and Haber-Bosch processes. The Bosch-Meiser process uses N_2_ as the nitrogen source, which is energy-intensive because the considerable dissociation energy (941 kJ/mol) is required to break the N≡N bond. Therefore, this technique is considered undesirable due to its high energy consumption, dependency on fossil fuels, and negative environmental impact.^7^^,^^8^

Today, the Haber-Bosch process is the most common method for NH_3_ production, employing principles similar to those of the Bosch-Meiser process but with further optimizations for greater efficiency and large-scale manufacturing. Nevertheless, the Bosch-Meiser process remains essential in advancing industrial NH_3_ production. Considering the mentioned drawbacks, it is suggested that the Haber-Bosch and Bosch-Meiser processes can be replaced with innovative, eco-friendly, and energy-efficient approaches for urea synthesis.^9^

Electrocatalysis, utilizing renewable electricity under milder conditions, has emerged as a promising, eco-friendly solution. It reduces the energy barrier of chemical reactions, providing substantial advantages and more efficiently addressing the practical challenges.^10^ Due to their cost-effectiveness and superior performance compared to conventional catalytic systems, electrochemical urea synthesis and production technologies have garnered considerable attention in scientific research and emerging markets.^11^ Electrocatalysis has also gained significant attention for sustainable urea synthesis. The main challenge is to catalyze efficient C–N coupling between carbon and nitrogen intermediates while minimizing undesired side reactions such as C–C coupling or H_2_ evolution.^12^^,^^13^ In this regard, electrocatalytic C−N bond coupling for converting CO_2_ and nitrogen sources (N_2_, nitrate (NO_3_^−^), nitrite (NO_2_^−^), NH_3_) into urea in ambient conditions has recently gained significant attention as a sustainable strategy for urea synthesis. However, traditional electrocatalysts have demonstrated limited performance in this area due to their poor chemical adsorption and weak surface C−N bond coupling.^14^

Therefore, ongoing research focuses on developing novel electroactive structures with enhanced physicochemical properties, optimized morphology, and improved electrochemical mass transfer to overcome these limitations in lab-scale projects. Various electrocatalytic structures, such as metallic alloys,^15^ mixed metal oxides,^14^ defective materials,^16^ organometallic structures,^17^ and semiconductors,^18^ have been widely explored to address this challenge, with a particular focus on improving selectivity as a key parameter in scaling up and industrializing the reaction. Photocatalytic C–N coupling is a promising, potentially green alternative strategy for synthesizing urea by directly coupling CO_2_ and nitrogen sources under light irradiation, typically using a semiconductor catalyst.^19^ This approach offers a safe, controllable, and environmentally sustainable route compared to traditional urea production methods, as mentioned before.^20^ The conventional urea synthesis process requires harsh reaction conditions (high temperature and pressure), making it a carbon-emitting, energy-intensive technology that contradicts carbon-neutrality goals. Whereas photocatalytic approaches can operate at ambient conditions, reducing both energy use and operational risk.^21^

Additionally, by utilizing sunlight as a renewable energy source and directly converting CO_2_, which helps in carbon capture and reduces greenhouse gas emissions,^22^ photocatalytic approaches offer a pathway for carbon-neutral production by directly transforming CO_2_ into valuable chemicals.^23^ Photocatalytic systems may also simplify process design by combining NH_3_ generation and urea formation in a single reactor.^24^ If catalysts are properly optimized, they can further limit byproducts and reduce environmental burden.^25^ Finally, semiconductor photocatalysts can be engineered to enhance selectivity, activity, and stability, allowing for optimized performance across a range of conditions.^26^ Despite these advantages, photocatalysts often suffer from high charge-carrier recombination rates and significant challenges in product controllability due to competing side reactions. To address these limitations and enhance overall performance, the methods of electrocatalysis and photocatalysis can be synergistically integrated using both light and electrical inputs, motivating the development of photoelectrocatalysts.

Over the past few decades, photoelectrocatalytic (PEC) technologies have demonstrated significant potential as sustainable solutions for clean energy production, utilizing methods such as water (H_2_O) splitting and CO_2_ reduction.^27^ These approaches have also been explicitly explored to enhance the yield and selectivity of organic synthesis reactions.^28^ Recently, researchers have focused on the efficient application of PEC methods to synthesize high-value chemicals, such as urea, through C–N coupling reactions on the surface of electrodes under light, taking advantage of the negligible external bias required and the virtually unlimited availability of solar energy.^29^ The key point in developing such an innovative tool is the strategic modification of the electrode surfaces with appropriate functionalities to catalyze the oxidation-reduction couplings, preventing the generation of undesirable byproducts. This review provides a comprehensive roadmap for advancing green urea synthesis by systematically comparing various techniques, including electrocatalytic, photocatalytic, and PEC methods, while emphasizing the synergistic potential of integrating PEC systems with electro- and photocatalysis as an underexplored strategy to enhance efficiency and sustainability in the field. Unlike previous studies, this review offers deeper mechanistic insights and a more comprehensive perspective by exploring various precursor mixtures, including CO_2_ + N_2_, CO_2_ + NO_3_^−^, and CO_2_ + NO, as well as the latest advancements in catalysts, such as Cu-TiO_2_, Cu_2_O, and Ru-doped systems. Additionally, it examines emerging approaches, including single-atom and hybrid catalysts. By going beyond the typical focus on catalysts and addressing critical challenges, such as low Faradaic efficiency, stability issues, and energy efficiency, it tries to bridge the gap between fundamental research and industrial scalability.

It also highlights gaps in urea quantification, emphasizing the need for standardized measurement protocols by addressing the analytical techniques, such as liquid chromatography-mass spectrometry (LC-MS), ultraviolet–visible spectrophotometry (UV-Vis), and nuclear magnetic resonance (NMR), essential for accurately measuring urea in catalytic systems. Furthermore, the research investigates the industrial feasibility of green urea production, comparing energy costs with traditional methods and discussing scalability, an aspect typically neglected in laboratory-focused reviews. Finally, it identifies research gaps and suggests future directions, particularly in optimizing PEC reactor design, offering a detailed guide that bridges laboratory advancements with practical, large-scale implementation.

## Electrocatalytic C–N coupling for urea synthesis

**Figure 1:**
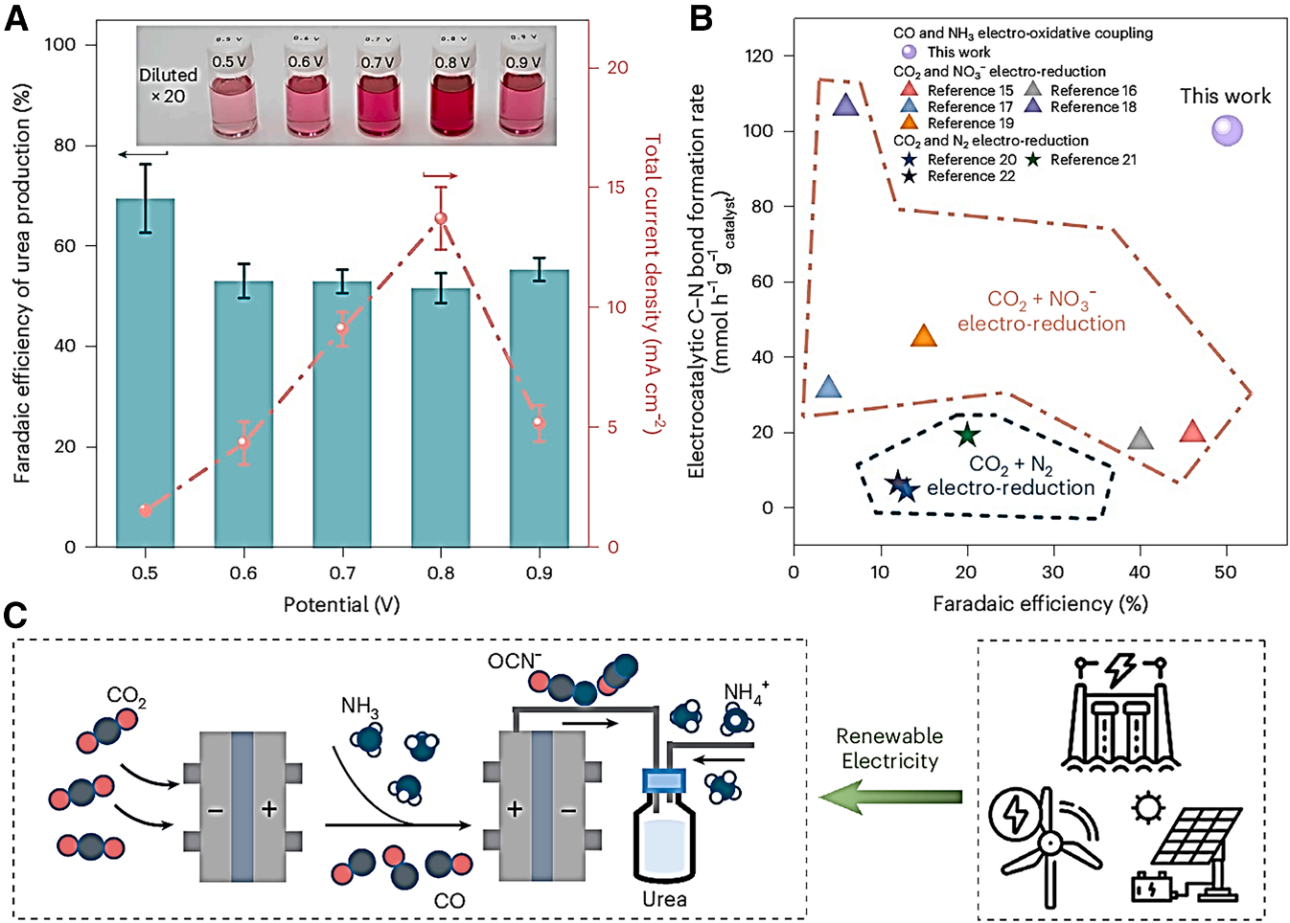
Electrocatalytic performance of urea synthesis on 70 wt.% Pt/C (A) Potential-dependent FE and total current density (the inserted photograph shows the post-reaction electrolytes after DAMO assays). (B) Comparison of the optimal electrocatalytic C–N bond formation rate and FE in this work, with reported values in the literature. (C) A schematic of the envisioned renewable electricity used for urea synthesis.^35^ Copyright 2024@Springer Nature.

Electrocatalytic C–N coupling has emerged as a promising approach for constructing C–N bonds and producing essential chemicals, such as urea, under mild ambient conditions.^13^ In contrast to the Haber–Bosch process, which relies on hydrogen produced via energy-intensive methods such as the H_2_O-gas shift reaction^30^ and steam–methane reforming,^31^ leading to significant CO_2_ emissions,^32^ electrosynthesis harnesses hydrogen directly from H_2_O, the most abundant resource on Earth.^33^ Therefore, in recent years, there has been significant interest in urea synthesis via electrochemical activation of various nitrogen precursors and CO_2_ on solid surfaces under mild conditions, thereby facilitating C–N coupling.^2^^,^^34^ Xiong et al. discovered a new electrocatalytic strategy for urea synthesis through the oxidative coupling of CO and NH_3_ on platinum (Pt) electrodes.^35^ Figure 1A shows that the total current density increases from approximately 1.5 to 13.7 mA cm^−2^ as the applied potential is raised from 0.5 to 0.8 V. Further increasing the potential to 0.9 V results in a decrease in current density to about 5.2 mA cm^−2^, indicating the onset of competing reactions or catalyst limitations. Notably, a high Faradaic efficiency for urea synthesis (∼70%) is achieved at 0.5 V, demonstrating excellent selectivity at lower potentials. As presented in Figure 1B, the electro-oxidative coupling of CO and NH_3_ over a commercial 70 wt. % Pt catalyst can deliver an impressive electrocatalytic C–N bond formation rate of 100 mmol h^−1^ g^−1^ at 0.8 V. According to Figure 1C, urea can be synthesized using renewable electricity through a two-step process in which CO_2_ is electrochemically reduced to CO at the cathode, after which the CO is electro-oxidatively coupled with NH_3_ at the anode of another reactor to form the urea. In this work, an efficient, alternative pathway for forming C–N bonds is demonstrated, avoiding challenges associated with reductive electrosynthesis, including hydrogen evolution.

The performance metrics of various electrocatalysts for urea synthesis, based on different carbon and nitrogen feedstocks, are presented in Table 1.

**Table 1:** Performance of various electrocatalysts for urea synthesis

Catalyst	Precursors (N- + C-source)	Applied potential (V vs. RHE)	Urea yield rate (μg/mg.h)	Faradaic Eff. (%)	Reference
Ru-Pd/WO_3_/MXene	N_2_ + CO_2_	−0.4	227	23.7	Govindan et al.^36^
Fe/MoS_2_	NO_3_^−^ + CO_2_	−0.5	18.98	54.98	Du et al.^37^
AuCu nanofiber	NO_2_^−^ + CO_2_	−0.3	215	18.2	Liu et al.^38^
Cu_99_Ni_1_	NO_2_^−^ + CO_2_	−0.6	361.3	25.1	Zhang et al.^39^
Cu–N–C–SACs	NO_3_^−^ + CO_2_	−0.5	120	28	Leverett et al.^40^
Vo-In-TiO_2_	NO_3_^−^ + CO_2_	−0.65	759.8	9.06	Mao et al.^41^
Cu_3_Mo_2_O_9_	NO_3_^−^ + CO_2_	−0.70	177	40	Zhang et al.^42^
Dynamic Bi nanosheets	N_2_ + CO_2_	−0.40	334.6	12.5	Wang et al.^43^
P–Cu/Fe_2_O_3_	NO_3_^−^ + CO_2_	−0.68	62.74	73.81	Deng et al.^44^
Cu^δ+^/Cu^0^	NO_3_^−^ + CO_2_	−1.2	386.4	25.2	Chen et al.^45^
FeNC-CeO_2_	NO_3_^−^ + CO_2_	−0.5	20969.2	89.3	Zhang et al.^46^
Diatomic Alloy catalyst CuPd_1_Rh_1_	NO_3_^−^ + CO_2_	−0.5	53.2	72.1	Chen et al.^47^
Cu1-MoS_2_	NO_3_^−^ + CO_2_	−0.6	23.3	57.02	Du et al.^48^

**Figure 2:**
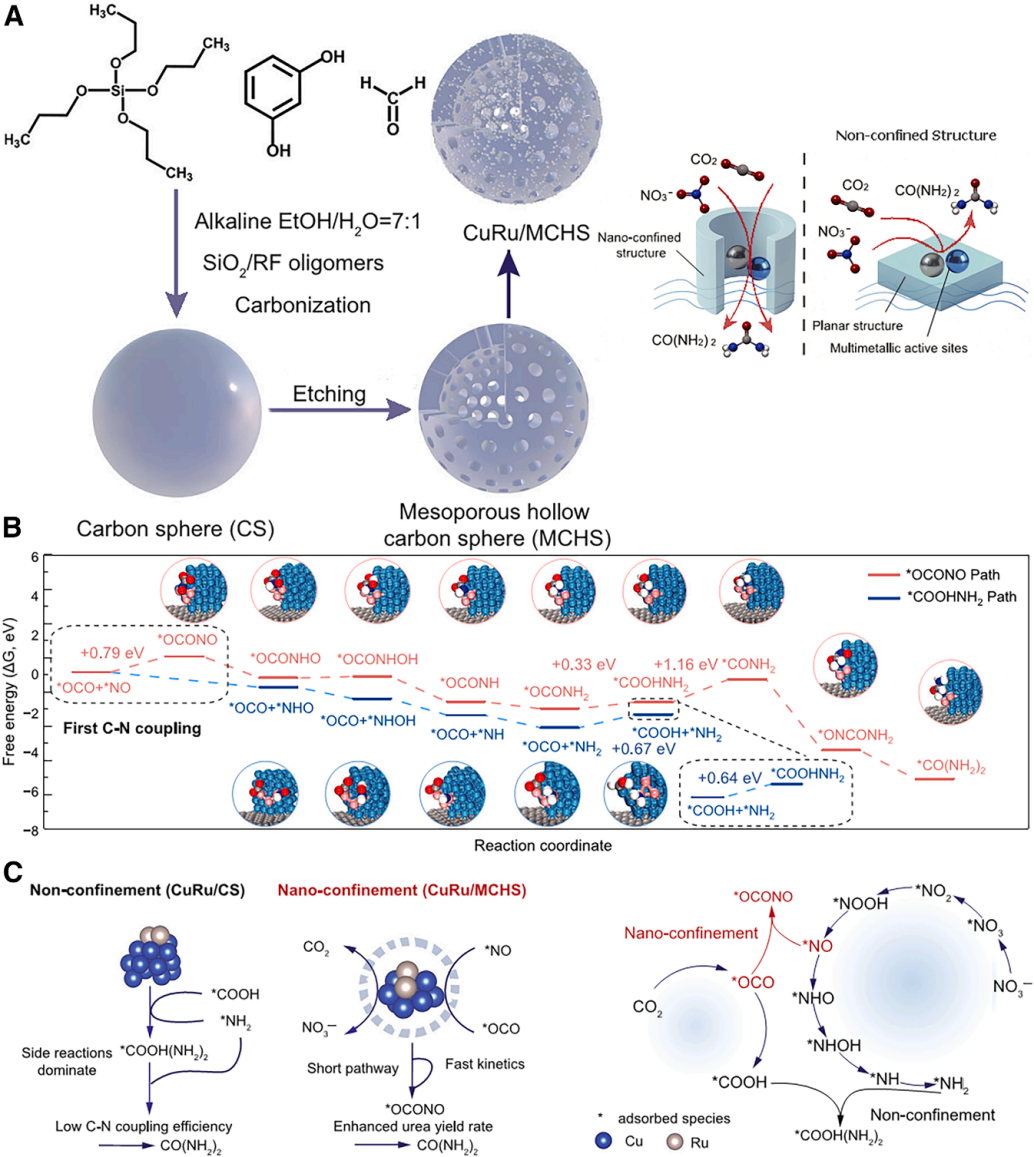
Structural characterization of CuRu/MCHS and CuRu/CS electro-catalyst (A) Synthesis process and microstructure of carbon spheres (CS), mesoporous carbon hollow spheres (MCHS), and CuRu/MCHS, alongside a schematic representation of the design principles of confined and nonconfined systems in urea electrosynthesis. (B) Reaction diagrams based on DFT calculations for urea production on the CuRu surface and corresponding atomic configurations. The gray, blue, red, white, pink, and turquoise spheres represent C, N, O, H, Cu, and Ru atoms, respectively. (C) Urea synthesis on CuRu/MCHS and CuRu/CS.^51^ Copyright 2025@Springer Nature.

Most of the above-mentioned systems suffer from low conversion efficiency and urea production yield, which remain substantial challenges in the field. These issues arise from the slow C–N coupling kinetics, limited CO_2_ adsorption capacity on metal catalysts, low reactant concentrations (CO_2_/NO_3_^−^) at high current densities due to poor mass transfer,^49^ and disordered adsorption/conversion processes that hinder essential intermediate interactions during urea formation.^50^ To improve catalytic efficiency and urea yield, it is crucial to enhance CO_2_/NO_3_^−^ adsorption, speed up their conversion, and stabilize major intermediates. A promising approach is the design of nano-confinement architecture, which spatially confines reaction pathways to specific domains, stabilizing reactive intermediates and controlling the dispersion of active components. Liu et al. synthesized mesoporous carbon hollow spheres (MCHS) and solid carbon spheres (CS) as catalyst supports to explore the impact of nano-confinement on C‒N coupling, employing Cu and Ru as bimetallic catalytic sites. Taking advantage of the CuRu synergy and nano-confinement effects, CuRu/MCHS achieved an impressive urea yield of 12.51 g h^−1^ g_cat_^−1^ at 250 mA cm^−2^, maintaining 125 h of stability (Figure 2A).^51^ To reveal the mechanistic differences between the nano-confined and non-confined systems, density functional theory **(**DFT) calculations were performed (Figure 2B). Adsorption energy analyses revealed that CO_2_ exhibited stronger bonding affinity with Cu (−1.58 eV) than Ru (−1.40 eV), whereas NO_3_^−^ preferentially interacted with Ru (−1.65 eV) rather than Cu (−1.12 eV). Based on the DFT results, they proposed two different pathways for C–N coupling in urea electrosynthesis, including (∗OCO + ∗NO → ∗OCONO) and (∗COOH + ∗NH_2_ → ∗COOHNH_2_). In the ∗OCONO pathway, the coupling requires an energy barrier of 0.79 eV. While the ∗COOHNH_2_ pathway exhibits a smaller energy barrier of 0.64 eV during the first C–N coupling step, it follows a much longer reaction path compared to the ∗OCONO pathway. After the initial C–N bond formation, the energy barrier for ∗CO_2_ hydrogenation is reduced from 0.67 eV (∗OCO + ∗NH_2_ → ∗COOH + ∗NH_2_) to 0.33 eV (∗OCONH_2_ → ∗COOHNH_2_), suggesting that urea formation becomes easier and faster after the first C–N coupling stage.

Figure 2C illustrates that the nano-confined structure of CuRu/MCHS enables early C–N coupling via the ∗OCONO pathway rather than the ∗COOHNH_2_ pathway typically followed in non-confined systems. Therefore, the final step for efficient urea production is the ∗OCONO pathway, where the coupling of ∗OCO and ∗NO to ∗OCONO initiates the process. The incorporation of Ru into the CuRu catalyst significantly lowers the energy barriers for this pathway, making it the preferred route for urea electrosynthesis while minimizing side reactions, such as HCOOH formation. The ∗OCONO pathway is more kinetically favorable, and nano-confinement helps break through the initial thermodynamic energy barrier, allowing the reaction to proceed more efficiently with fewer side reactions. The diagram shows how nano-confinement stabilizes intermediates, thereby enhancing the overall efficiency of urea electrosynthesis by shifting the coupling reaction toward a faster and more effective pathway.

Despite the increasing number of reports in this field, the topic continues to face significant challenges, including an unclear mechanistic description of the processes, low production yield, and poor Faradaic efficiency, which severely hinder its extensive use in practical applications.^52^ In addition, due to the complex multi-electron pathway involving a variety of intermediates, the reaction kinetics are slowed, and more energy is required to achieve practical current densities than expected.^53^ Considering this, achieving selective C–N coupling is essential, and understanding the underlying reaction mechanisms is crucial for designing efficient electrocatalysts capable of precise and controllable C–N bond formation.^54^ Electrocatalytic C–N coupling involves the simultaneous electrochemical activation of carbon-containing reactants, such as CO_2_, and nitrogen-containing compounds, such as NH_3_, nitric oxide (NO), NO_2_^−^, and NO_3_^−^, with both reacting at the same electrode surface. Depending on the electrochemical environment, these reactions are either reductive cathodic or oxidative anodic C–N couplings.^55^ In cathodic C–N coupling reactions, C–N bonds are formed through electrochemical reduction processes occurring at the cathode surface, while anodic C–N coupling involves the formation of carbon–nitrogen bonds through oxidative electron transfer processes occurring at the anode surface.^56^ The selection of reactants and the characteristics of intermediate species play a crucial role in determining reaction pathways and product selectivity.^57^ The wise choice of reactants is key to directing the formation of desired C–N coupling products, making it essential for precise and efficient organic synthesis.^58^

### Mechanism of urea synthesis from CO_2_ and nitrogenous sources

Several types of nitrogen species, including NO_2_^−^,^59^ NO_3_^−^,^40^ molecular N_2_,^15^ and NH_3_^60^ have been widely investigated for electrocatalytic coupling with CO_2_ on electrode surfaces to produce urea. A thorough understanding of the reaction mechanisms involving these precursors is critical to optimizing efficiency under ambient conditions. Beyond theoretical studies employing established methods, such as DFT, advanced *in situ* spectroscopic techniques have proven instrumental in elucidating these pathways. For instance, Raman spectroscopy, synchrotron radiation Fourier transform infrared spectroscopy (SR-FTIR), and sum-frequency generation infrared spectroscopy (SFG-IR) have been successfully applied to unravel the mechanistic details of electrocatalytic urea synthesis from these nitrogen-containing compounds.^2^^,^^52^

#### Urea formation from carbon dioxide and nitrogen

Both N_2_ and CO_2_ are chemically inert due to their strong bonding energies of 941 kJ mol^−1^ for N≡N and 806 kJ mol^−1^ for C=O, making their activation challenging,^61^ while electrocatalysts facilitate the activation of these molecules, enabling catalytic reactions under milder conditions.^62^ However, competing reduction processes of CO_2_ to CO, formate, or methane and N_2_ to NH_3_ or hydrazine (N_2_H_4_) hinder efficient C–N coupling and often lead to low urea selectivity.^15^^,^^63^ Recent advancements have focused on overcoming these limitations by strategically designing and developing tailored electrocatalysts.^64^ Govindan et al. developed a series of bifunctional heterojunction electrocatalysts by anchoring Ru-Pd alloy nanoparticles onto 2D MXene and WO_3_ nanosheets (Ru-Pd–WO_3_/MXene) for urea synthesis via the electrochemical coupling of CO_2_ and nitrogen.^36^ According to their results, the optimum catalyst achieved a Faradaic efficiency of 23.7% and an excellent urea production yield of 227 μg_urea_ mg_cat_^−1^ h^−1^. The C–N coupling mechanism for urea formation was further investigated using DFTcalculations, which proposed that the reaction is initiated by the reduction of CO_2_ to adsorbed ∗CO on the electrode surface. This intermediate then reacts with nitrogen to form ∗NCON, which subsequently undergoes protonation to yield urea.

To efficiently promote the urea formation reaction, anodic oxidation of H_2_O to enrich the medium with H^+^ and (e^−^) is crucial. During this process, two H_2_O molecules are oxidized at the Ru-Pd anode, generating substantial amounts of hydronium ions (H_3_O^+^) and electrons, as well as CO_2_ and NO_3_^−^ species. Equations 1, 2, 3, 4, 5, 6, and 7 describe the proposed mechanism of the urea formation reaction over the catalyst mentioned above.

Anode:

(Equation 1) $$2H_{2}O\to O_{2}+{4H}^{+}+{4e}^{-}$$

Cathode:

(Equation 2) CO2+∗+e−→CO2−∗

(Equation 3) CO2−∗+M+→CO2−∗−M+

(Equation 4) CO2−∗+M++H2O→COOH∗+M++OH−

(Equation 5) COOH∗+e−→COOH∗+OH−

(Equation 6) CO∗+N∗2→NCON∗→NCONH∗→NHCONH∗→NHCONH2∗→NH2CONH2∗

Overall:

(Equation 7) $$N_{2}+{\text{CO}}_{2}+{6H}^{+}+{6e}^{-}\to H_{2}O+NH_{2}\text{CON}H_{2}$$

**Figure 3:**
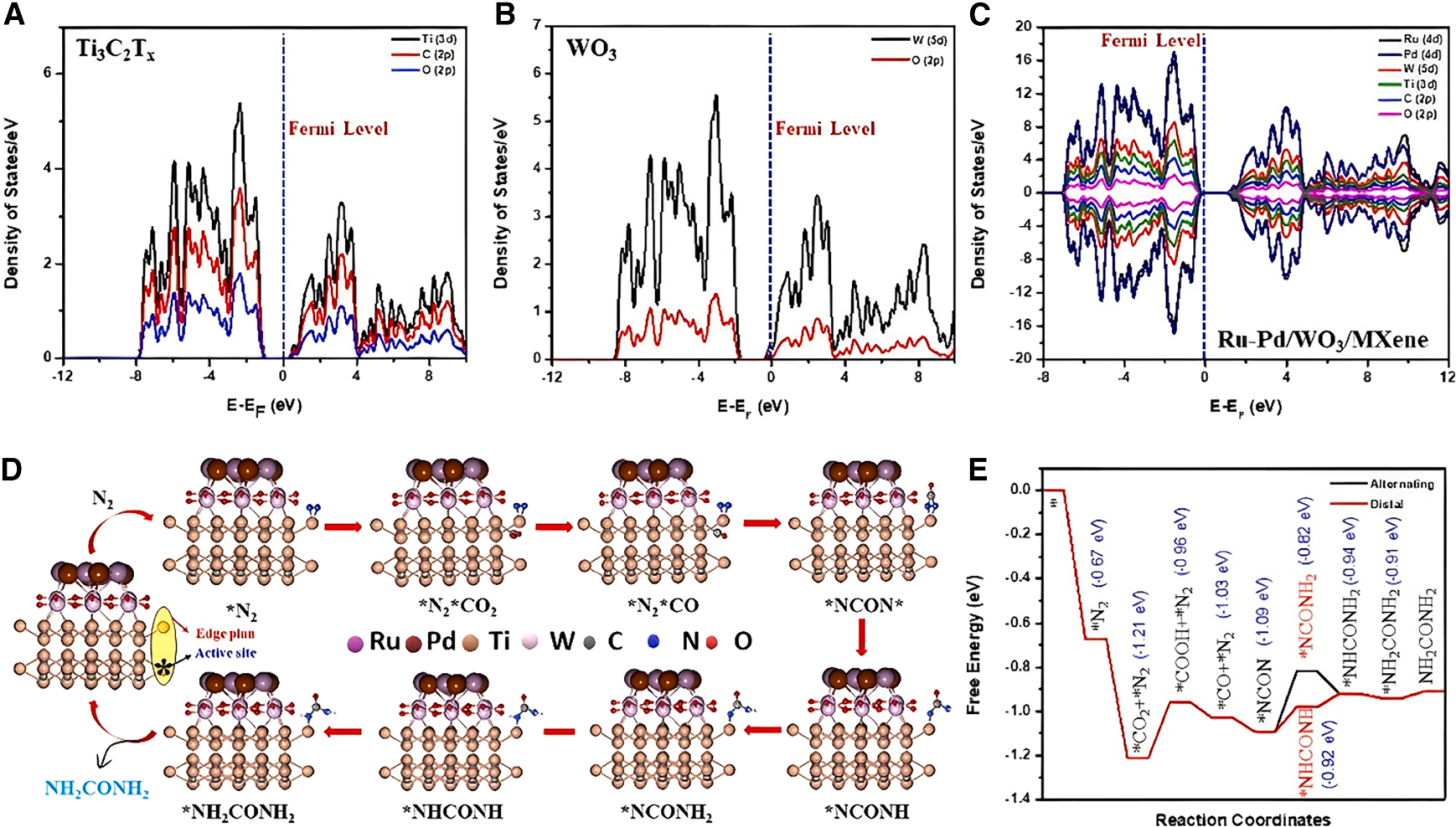
A DFT study was conducted to investigate Ru–Pd/WO_3_/MXene for accelerated CO_2_N_2_RR DOS of Ti_3_C_2_Tx (A), WO_3_ (B), and Ru−Pd/WO_3_/MXene (C), mechanism of N_2_-CO_2_ reduction on the prepared catalyst (D), and free energy diagram for the reaction through an alternative and distal pathway on Ru−Pd/WO_3_/MXene (E).^36^ Copyright 2024, American Chemical Society.

DFT results for the photoelectrochemical reduction of CO_2_ and N_2_ to urea indicate that Ru–Pd/WO_3_/MXene exhibits a stronger tendency for the chemisorption of N_2_ compared to pure MXene or WO_3_. The results also confirmed that incorporating Ru-Pd into the WO_3_/Mxene structure can remarkably shift the density of states (DOSs) toward the Fermi level, indicating a substantial improvement in the electrical conductivity of the material (Figures 3A–3C). Based on the initial stage of the PEC process and free energy diagram of the reduction reaction (Figures 3D and 3E), an excellent urea yield could be achieved at low onset potential over the prepared bifunctional surface compared to the recently reported Pd-containing heterostructures, such as Pd–CeO_2_^20^ and Pd_1_Cu_1_/TiO_2_-400.^15^ The enhanced selectivity of the catalyst results from a carefully designed multifunctional heterostructure, in which each component plays a specific role in supporting distinct steps of the C–N coupling process. Ru–Pd alloy nanoparticles activate CO_2_ and convert it to ∗CO, with the bimetallic combination lowering the energy barrier for this key intermediate. WO_3_ nanosheets, with a low conduction band, weaken the N≡N bond to activate ∗N_2_, while MXene (Ti_3_C_2_T_x_) provides a highly conductive surface, adsorbing and activating N_2_ and preventing nanoparticle clumping. This 2D-on-2D architecture enhances charge transfer and creates a favorable electronic environment for C–N coupling between ∗CO and ∗N_2_, forming ∗NCON. The design minimizes competing reactions by inhibiting hydrogen evolution, guiding ∗CO toward C–N coupling, and preventing NH_3_ formation through controlled N_2_ activation.

Similar theoretical and experimental insights have been reported by other researchers investigating Zn-Mn diatomic catalyst structures^65^ and Ti_2_@C_3_N_4_.^66^ All findings are based on the understanding that the chemical adsorption of the reactants is a key step in the reaction, driven by interactions between N_2_ and/or CO_2_ and the metallic active sites on the electrocatalyst components. Another DFT study on CuB_12_ single-layer structures revealed that the CuB_12_ surface exhibits excellent catalytic performance along with the CO_2_ and OCOH pathways, with a low limiting potential of approximately 0.23 V and a low kinetic barrier of 0.54 eV for the key C–N bond-forming step (∗CO + ∗NHNH → ∗NHCONH), indicating strong intrinsic activity and high selectivity toward urea formation. The high selectivity of the CuB_12_ monolayer arises from its electron-deficient boron sites, which strongly adsorb and activate both CO_2_ and N_2_ while stabilizing key intermediates such as ∗CO and ∗NHNH in close proximity. Selectivity is further enhanced by suppressing CO_2_ reduction pathways leading to CH_3_OH or CH_4_, as further hydrogenation of ∗CO to ∗CHO is thermodynamically and kinetically less favorable than C–N coupling. In parallel, the reaction mechanism bypasses complete N_2_ reduction to NH_3_ by promoting coupling between CO and partially hydrogenated N_2_ species, preventing NH_3_ from becoming a dominant nitrogenous by-product, even though the initial N_2_ activation step shares a similar energy barrier. Additionally, weak hydrogen adsorption on boron sites intrinsically suppresses the hydrogen evolution reaction, ensuring that protons are preferentially consumed in urea-forming hydrogenation steps rather than H_2_ generation. Together, these features enable the CuB_12_ catalyst to efficiently steer reaction pathways toward selective urea production.^67^

#### Urea formation from carbon monoxide and ammonia

Direct C–N coupling between CO and NH_3_ is challenging due to the inert nature of CO; however, under suitable catalytic conditions, this barrier can be overcome by activating the CO molecule, thereby facilitating the coupling reaction toward urea formation.^35^ The electrocatalytic C–N coupling of CO and NH_3_ on noble metal catalysts has recently been investigated to elucidate the reaction mechanism, employing electrochemical and computational techniques coupled with *in situ* spectroscopic analysis, particularly nuclear magnetic resonance (NMR).^35^ The process demonstrated a strong performance on Pt catalysts, achieving approximately 70% selectivity and a C–N bond formation rate of 100 mmol h^−1^ g^−1^ at ambient conditions. The proposed reaction mechanism also involves sequential 2H^+^-coupled electron-transfer steps leading to the formation of the intermediate isocyanic acid (HNCO). The HNCO intermediate was subsequently neutralized to form cyanate, which then reacted with ammonium to yield urea. The practical significance of this method lies in its efficient utilization of toxic CO gas to synthesize high-value-added urea through coupling with nitrogen, an abundant atmospheric component.

#### Urea formation from carbon dioxide and nitrites

Previous studies suggested that the C–N coupling step during urea electrosynthesis from NO_2_^−^ and CO_2_ likely originates from ∗NH_2_ and ∗CO intermediates.^68^ A possible mechanism for this reaction was proposed by Wang’s research team, considering their experimental results for electrocatalytic urea generation on the surface of the AuCu self-assembled nanofiber catalysts^38^:

(Equation 8) CO2+2H++2e−→CO∗+H2O

(Equation 9) NO2−+6H++5e−→NH∗2+2H2O

(Equation 10) CO∗+2NH∗2→NH2CONH2

The proposed pathway involved the simultaneous formation of ∗CO and ∗NH_2_ intermediates on electrode surfaces, followed by their coupling to form urea. The authors also clearly claimed that no urea formation was observed when CO_2_ and NO_2_^−^ were replaced with CO and NH_4_^+^, respectively. Another team reported a similar mechanistic trend for the reaction over a Cu_99_Ni_1_ catalyst, supported by DFT simulations and *in situ* FTIR spectroscopy.^69^ However, a key deviation from the standard NH_3_-based pathway was observed in the work of Zhang et al., where the oxygen vacancies in ZnO play a role by providing active sites for the direct C–N coupling of CO_2_ and NO_2_^−^ derivatives. According to their results from *in situ* ATR (attenuated total reflectance)-FTIR spectroscopy, the electrochemical generation of ∗NH_2_ and ∗COOH via proton-coupled electron transfer steps represents an initial key step in the urea formation pathway. This claim was verified by the detection of their characteristic peaks in ATR-FTIR spectra.

(Equation 11) NO2−+6H++5e−→NH∗2+4H2O

(Equation 12) CO2+e−+H+→COOH∗

(Equation 13) COOH∗+2NH∗2+H++e−→NH2CONH2+CO+H2O

#### Urea formation from carbon dioxide and nitrates

There is a global concern about NO_3_^−^ content in groundwater, primarily due to the excessive use of fertilizers and other industrial activities.^70^ Given that the N=O bond (204 kJ mol^−1^) has a substantially lower dissociation energy than the N≡N bond (941 kJ mol^−1^), electrocatalytic reduction of NO_3_^−^ has emerged as an attractive and environmentally friendly approach, particularly when coupled with CO_2_ co-reduction, enabling urea synthesis via the C–N coupling process.^71^ Considering such advantages along with the growing interest in sustainable nitrogen and carbon utilization, recent research has increasingly focused on CO_2_ and NO_3_^−^ as commonly used reactants in electrocatalytic urea synthesis. This focus results from the fact that these reactants are abundant, sustainable, and allow direct carbon-nitrogen coupling under mild conditions.^72^ The efficiency and pathway of urea formation, however, strongly depend on the choice of nitrogen precursor and its oxidation state. As a deeply oxidized species, NO_3_^−^ undergoes a more complex reduction process than NO_2_^−^, as in many studies with NO_3_^−^ and CO_2_, the C–N bond forms via ∗NO_3_, ∗NO_2,_ and ∗CO/∗CO_2_.^73^ The series of reactions outlined in Equations 14, 15, 16, 17, 18, 19, 20, and 21 describes a rational mechanism for urea formation. NO_3_^−^ ions are first reduced to ∗NH_2_^−^ on the catalyst surface. This reaction involves the consumption of protons (H^+^) and electrons (e^−^), leading to the formation of ∗NH_2_ and H_2_O.

(Equation 14) NO3−+2H++2e−→NO2−∗+H2O

(Equation 15) NO2−∗+2H++2e−→NO−∗+H2O

(Equation 16) NO−∗+4H++4e−→NH2∗+H2O

Overall, NO_3_^−^ is reduced to an amine group (∗NH_2_) on the catalyst surface (∗), with the release of H_2_O:

(Equation 17) NO3−+8H++8e−→NH2∗+3H2O

DFT exploration shows that ∗NH_2_, as a reactive nucleophile, is kinetically accessible and stable on transition metal surfaces.^74^

(Equation 18) CO2+e−+H+→COOH∗

(Equation 19) COOH∗+H++e−→CO∗+H2O

∗COOH is a transient intermediate, while ∗CO is the stable surface species that is adsorbed strongly enough to exclude further reduction of CO_2_.^75^

(Equation 20) CO∗+NH2∗+H++e−→CONH2∗+CO+H2O

DFT calculations also demonstrated a lower formation energy for ∗CONH_2_ on Te−Pd (111) than that of pure Pd.^76^

(Equation 21) CONH2∗+NH2∗+H++e−→NH2CONH2

**Figure 4:**
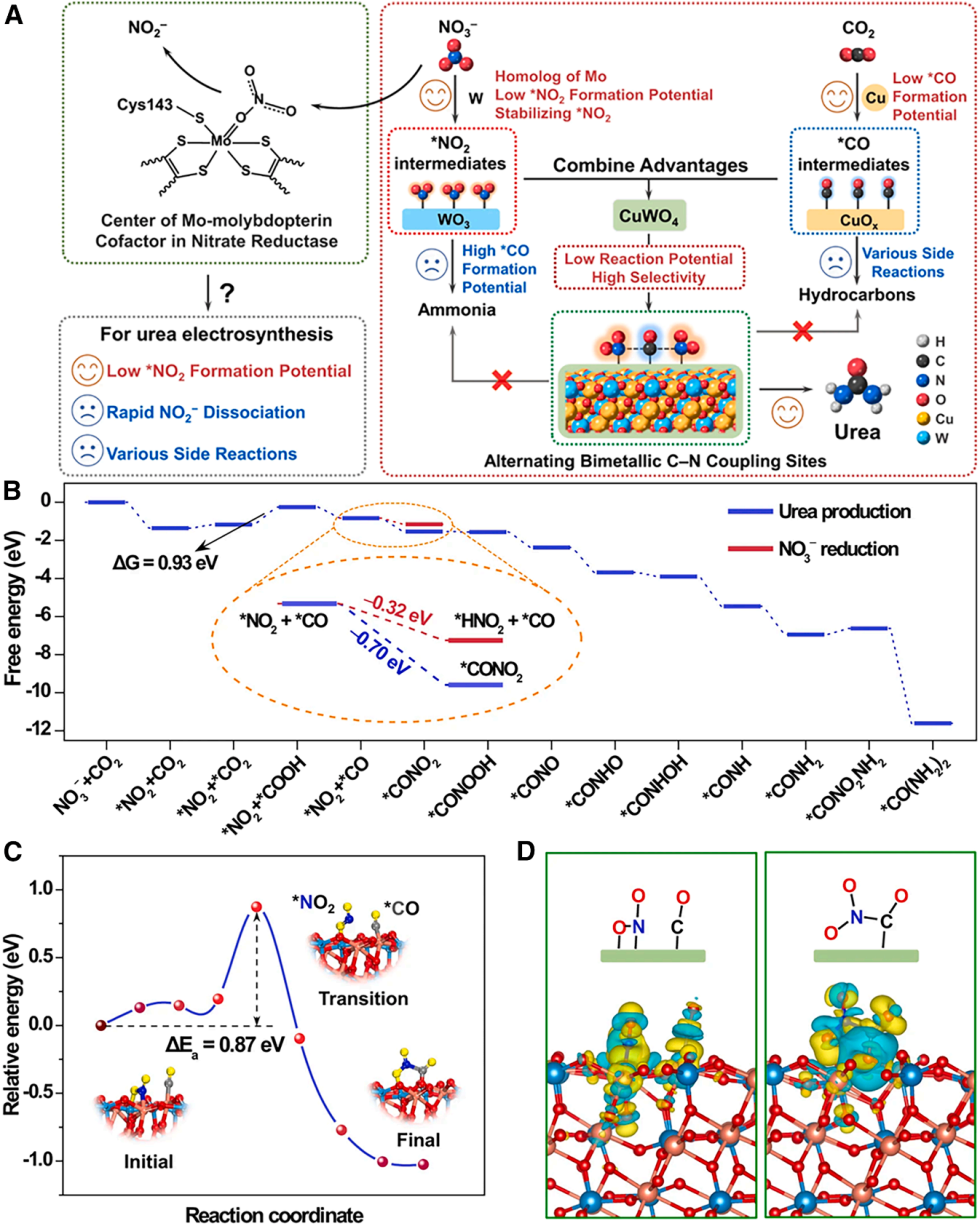
Schematic illustration of design strategy for bioinspired alternating bimetallic sites of CuWO_4_ catalyst for urea electrosynthesis and DFT calculation of urea synthesis mechanism on bimetallic CuWO_4_ (111) surface (A) CuWO_4_ catalyst design, showing bioinspired alternating bimetallic sites for urea electrosynthesis. (B) Free-energy diagram illustrating urea formation and NO_3_^−^ reduction on the CuWO_4_ (111) facet. (C) Mechanism of C–N coupling between ∗CO and ∗NO_2_, showing the initial, transition, and final states of ∗CONO_2_ formation. Atom colors: gray = C, blue = N, red = lattice O, orange = Cu, cyan = W, yellow = adsorbate O. (D) Charge density difference for the co-adsorption of ∗CO and ∗NO_2_ (left) and for the adsorbed ∗CONO_2_ (right) on the CuWO_4_ (111) facet. The iso-value is 0.002 e/Å^3^, with electron accumulation shown in yellow and depletion in cyan.^78^ Copyright@2023, Springer Nature.

To elucidate the mechanism of C–N coupling during the co-reduction of NO_3_^−^ and CO_2_, the online differential electrochemical mass spectrometry (DEMS) technique was applied. Meng et al. demonstrated that ∗NH_2_ and ∗CO are the primary intermediates in the reaction mechanism over Cu-Zn alloy, as indicated by the significant decrease in the signal intensity of CO and NH_2_ upon the consumption of the reactants.^77^ Further mechanistic insights were obtained by investigating the energy barriers associated with forming key intermediates on various metal facets, including Zn and Cu-Zn alloys. Despite these findings, many studies still suggest that ∗NO_2_ and ∗CO/∗CO_2_ are the dominant intermediates in NO_3_^−^-to-urea conversion. Zhang et al. introduced copper molybdate (Cu_3_Mo_2_O_9_) nanorods as an active hydrogen pump catalyst that actively generates and supplies surface-adsorbed hydrogen (∗H) at Cu sites to hydrogenate key nitrogen and carbon intermediates. In the nitrogen-converting route, ∗NO is rapidly converted to ∗HNO and then to ∗NH_2_O or ∗NHOH, ultimately yielding the ∗NH_2_ intermediate. In the carbon conversion pathway, CO_2_ is converted to ∗COOH, and then hydrogenated to the ∗HOCOH intermediate, which is more electrophilic than ∗CO and favors nucleophilic attack by ∗NH_2_. The C–N coupling occurs between ∗NH_2_ and ∗HOCOH, forming ∗H_2_NCOOH, which, upon dehydration, gives ∗H_2_NCO and is finally hydrogenated to urea. The synergistic effect of Cu sites for ∗H generation and NO_3_^−^/NO_2_^−^ activation, together with Mo-O motifs that stabilize intermediates and modulate electronic structure, suppresses side reactions and enables the formation of a new, lower-energy pathway via hydrogenated intermediates.^42^ Using a combination of DFT calculations, DEMS, temperature-programmed desorption (TPD), and *in situ* Raman spectroscopy, Zhao et al. identified the rate-determining step and the prominent intermediates.^78^ They designed an alternating Cu–W bimetallic catalyst (CuWO_4_) with high-valence W^6+^ sites specifically tailored to stabilize ∗NO_2_ intermediates from NO_3_^−^ reduction, while adjacent Cu^2+^ sites favor the formation of ∗CO intermediates from CO_2_ reduction at remarkably low potentials (Figure 4A). This wise arrangement brings the carbon and nitrogen intermediates close together, dramatically increasing the probability of direct C–N coupling. The prepared CuWO_4_ demonstrates outstanding performance, achieving a urea Faradaic efficiency of 70.1 ± 2.4% and a production rate of 98.5 ± 3.2 μg h^−1^ mg^−1^ at a low potential of −0.2 V versus RHE, significantly exceeding both its single-metal counterparts (CuO and WO_3_) and previous state-of-the-art catalysts.^78^ A comprehensive mechanistic study, using *in situ* Raman spectroscopy, DEMS, and TPD, confirmed that ∗CO and ∗NO_2_ are the key surface-bound intermediates. Importantly, changing the reactants in the experiments proved that only CO (or its aqueous derivatives) and NO_2_ gas could replace CO_2_ and NO_3_^−^ to produce urea, confirming their roles as the essential coupling partners. DFT calculations provided an atomic-level understanding, showing that the rate-determining step is the C–N coupling between ∗CO and ∗NO_2_ to form ∗CONO_2_ (Figures 4B–4D) This coupling is thermodynamically more favorable (ΔG = −0.70 eV) than the competing hydrogenation of ∗NO_2_ to NH_3_ (ΔG = −0.32 eV) or ∗CO to hydrocarbons, which explains the reason for the high selectivity of the system. The electronic structure at the bimetallic interface promotes electron transfer, facilitating this key coupling step. Therefore, this study successfully highlights a general design principle by constructing catalysts with complementary, well-arranged sites that stabilize and bring key intermediates together, and effectively control complex reaction pathways. This strategy minimizes dominant side reactions, such as NH_3_ or hydrogen evolution, and enables highly selective and efficient synthesis of urea from abundant feedstocks, such as CO_2_ and NO_3_^−^.

Moreover, by testing alternative carbon sources (CO_3_^2−^, HCO_3_^−^, CO, HCHO, CH_3_OH) while keeping the nitrogen source constant, the authors demonstrated that only precursors that form the adsorbed ∗CO intermediate can lead to urea, confirming ∗CO as the essential carbon partner. In parallel, screening different nitrogen sources (NO_2_, NO_2_^−^, NO, NH_2_OH, NH_3_) with CO_2_ revealed that only NO_2_ gas could substitute for NO_3_^−^, ∗NO_2_ as a nitrogen intermediate. This highlights the crucial role of precursor selection, which dictates which nitrogen intermediates are generated and how effectively they couple with carbon species. Choosing an appropriate nitrogen source for urea synthesis is inherently complex because it requires balancing thermodynamic feasibility, kinetic behavior, and practical system constraints. No single option is universally superior, as the choice fundamentally shapes the overall process design. Although NO_3_^−^ is thermodynamically favorable based on standard potential, it demands the highest proton consumption per urea molecule. From a thermodynamic perspective, NO is favorable when compared to the competing anodic H_2_O-splitting reaction, as it shows the smallest change in Gibbs free energy per urea molecule. In terms of kinetics, NO_2_^−^ and NO provide higher current densities than nitrogen gas. This is consistent with the previous electrochemical observations, in which N_2_ behaves inertly compared to other nitrogen sources. Practically, N_2_ and NO are gases, while NO_3_^−^ and NO_2_^−^ are dissolved in the electrolyte, allowing independent control over nitrogen and CO_2_ transport. Regardless of the nitrogen source, a liquid phase is required in the cell, necessitating carefully designed zero-gap configurations to minimize voltage. Although NO_3_^−^, NO_2_^−^, and NO are more reactive, they are less abundant than N_2_, a key consideration for real-world applications and the overall feasibility of the process.^79^ For instance, pursuing N_2_ activation aligns with long-term sustainability goals when abundant renewable energy is available, whereas short-term, practically deployable electrosynthesis is more effectively achieved with NO_3_^−^/NO_2_^−^ reactants, especially when paired with highly active electrocatalysts capable of transforming these pollutants into useful products.^79^ Table 2 summarizes various nitrogen precursors for urea synthesis, outlining their respective advantages and challenges, thereby linking experimental evidence to strategic catalyst and reactant design.

**Table 2:** Nitrogen precursors for urea synthesis: advantages and disadvantages

Nitrogen precursor	Activation difficulty	Nitrogen intermediates	Advantages	Challenges	Reference
N_2_ (Nitrogen gas)	very high	∗N_2_, ∗NH_2_, ∗NCON, ∗NNH	abundant, clean, sustainable	extremely difficult activation due to a strong N≡N bond; very low Faradaic efficiency; requires harsh conditions	Chen et al.^15^; Foster et al.^80^
NO_3_^−^ (Nitrate ion)	moderate	∗NO_3_, ∗NO_2_, ∗NO, ∗NH_2_	high solubility; good selectivity when ∗NO_2_ stabilizes, widely available	multiple reduction steps; competing NH_3_ formation; requires precise control of intermediates	Yu et al.^81^; Zeng et al.^82^
NO (Nitric oxide)	low-moderate	∗NO, ∗NO_2_, ∗N, ∗NH_2_CO	easy activation; strong N–metal interaction; rapid formation of urea intermediates	toxic; unstable; undesired side reactions; handling difficulties	Cai et al.^83^; Long et al.^84^; Shi et al.^85^
NO_2_^−^ (Nitrite ion)	low	∗NO_2_, ∗CO_2_, ∗NOOH, ∗NH_2_	high urea yield; low activation energy; low N=O bond dissociation energy; water-soluble	competing NH_3_ formation; mass-transport sensitive; selectivity depends on catalyst	Hu et al.^86^; Shin et al.^87^; Cai et al.^83^
NH_3_/NH_4_^+^ (Ammonia)	very low	∗NH_2_, ∗NH, ∗NCON	easy to activate; simple chemistry; can couple electrochemically with ∗CO	primarily produces H_2_ or reduces to N_2_; not ideal for direct C–N coupling; low selectivity for urea	Krebs et al.^88^; Krase et al.^89^

#### Mechanistic divergence and the catalyst-dependent pathway

As discussed in the previous sections, the literature reports several conflicting C–N coupling pathways, including the coupling of ∗CO and ∗N_2_ to form an ∗NCON intermediate (Equation 6), the reaction of ∗COOH and ∗NH_2_ intermediates (Equation 13), and the coupling of ∗CO and ∗NO_2_ according to Zhao et al.’s study.^78^ This review argues that such divergence does not reflect errors in analysis; rather, it represents a significant insight that the urea synthesis mechanism is highly catalyst-dependent and not universal. The nature of the active sites, whether a metal alloy, metal oxide, or single-atom catalyst (SAC), along with the specific nitrogen precursor (N_2_ versus NO_3_^−^), fundamentally determines which intermediates are energetically favored and, therefore, which reaction pathway predominates. For instance, in the Ru-Pd/WO_3_/MXene heterostructure, the Ru sites provide strong N_2_ binding that facilitates N_2_ activation, while the Cu or Pd sites favor CO_2_ reduction to ∗CO. This dual functionality facilitates the coupling of nitrogenous and carbonaceous intermediates via the ∗NCON pathway (Equation 29), which is energetically plausible. Therefore, cooperative interactions between different sites within a heterostructure play a critical role.

As discussed in urea formation from CO_2_ and NO_3_^−^, the choice of nitrogen precursor is crucial, as it fundamentally alters the reaction pathway. Table 2 demonstrates that N_2_ is highly inert, requires significant energy for activation, and typically undergoes associative or dissociative reduction via ∗N_2_ or ∗NH_2_ intermediates. In contrast, NO_3_^−^, as an oxidized and solvated species, is reduced through intermediates such as ∗NO_2_, ∗NO, and ∗NH_2_.

On the other hand, oxide-based catalysts with abundant oxygen vacancies, such as ZnO or Vo–In–TiO_2_, readily activate H_2_O to supply protons and preferentially stabilize ∗COOH (from CO_2_) and ∗NH_2_ (from NO_2_^−^ reduction). For these systems, the ∗COOH + ∗NH_2_ coupling pathway (Equation 13) is therefore the more probable route.

In the PEC systems, light and an applied electrical bias operate as distinct synergistic parameters that together drive chemical reactions, each shaping catalyst behavior and reaction mechanisms in fundamentally different ways. Light primarily generates electron–hole pairs within a semiconductor when photon energy exceeds the bandgap, creating high-energy charge carriers, altering surface potentials, and sometimes restructuring the catalyst surface. In contrast, the applied electrical bias controls the separation and utilization of these photogenerated charges by establishing the electric field, setting the Fermi level, and tuning the overpotential, thereby affecting the binding energies and reaction pathways of surface intermediates. Their interaction creates a unique operational regime in which light supplies part of the reaction energy while the bias directs charge flow and sets the thermodynamic driving force, enabling mechanistic pathways not accessible in purely electrochemical or purely photocatalytic systems. Consequently, reaction performance reflects a non-linear combination of light intensity and applied potential, implying that the selectivity and activity depend sensitively on how these two inputs are jointly tuned.

The absence of a single, universal mechanism underscores the need for future research to move beyond merely proposing mechanistic pathways and instead prioritize catalyst designs that both minimize the energy barrier for a targeted, high-selectivity route and concurrently elevate the kinetic barriers for all competing parasitic reactions.

### Electrocatalytic materials for sustainable urea production

As mentioned in the previous sections, CO_2_ and nitrogenous compounds, such as N_2_, NO_2_^−^, NO_3_^−^, and NO, are the primary reactants for urea production under electrocatalysis conditions. One key challenge in this approach is achieving the controlled, simultaneous reduction of CO_2_ and N-containing species within the same reaction environment. Therefore, the rational design of advanced electrocatalytic materials to control reaction selectivity and enhance urea yield is a key point in this field. Considering several crucial factors, including the mass transfer rate at the catalyst surface/interface, the number of accessible active sites, and the electronic behavior at the catalyst–electrode interface, various solid materials have been explored for efficient urea electrosynthesis over the past decades.^90^ In this section, the focus is on the primary principles and essential factors in designing rational electrocatalysts for efficient urea production, as well as the remarkable challenges that restrict their large-scale use in real-world applications.

#### Transition metals

Due to the unique properties such as high stability, low cost, abundance in nature, desirable electronic characteristics, and environmental friendliness, transition metals such as Zn, Fe, Cu, Co, V, Mo, Ce, W, and others have received increasing attention for promoting the electrocatalytic synthesis reactions.^14^^,^^66^^,^^91^^,^^92^^,^^93^^,^^94^ Precise engineering of structural characteristics and atomic-level morphology significantly influences the electrochemical performance and catalytic activity of transition-metal-based materials. As a result, considerable effort has been devoted to designing and fabricating SACs in recent years to enhance the electrocatalysis of CO_2_ and nitrogenous compound reduction, NH_3_ synthesis, hydrogen evolution, and C–N coupling reactions.^2^ The SACs, which consist of individually isolated atoms, offer high atom efficiency and well-defined active sites, making them highly effective for catalytic C–N coupling reactions. Fe-based SACs have demonstrated the capability to simultaneously activate N_2_ and CO_2_, thereby facilitating C–N coupling, which is essential for urea formation. Du et al. synthesized Fe single atoms supported on MoS_2_ (Fe_1_/MoS_2_) as effective catalysts for urea electrosynthesis.^37^ This catalyst achieves a Faradaic efficiency of 54.98% and a urea yield of 18.98 mmol h^−1^ g^−1^, in a flow cell, which is attributed to a well-designed tandem catalytic mechanism in which Fe_1_–S_3_ sites preferentially drive the initial C–N coupling while MoS_2_ edge sites facilitate the subsequent hydrogenation of the ∗CO_2_NH_2_ intermediate to ∗COOHNH_2_ and thereafter urea. Although the results are scientifically impressive, reproducing them may be challenging because the formation of the Fe_1_–S_3_ active sites is highly sensitive to synthesis conditions, and their verification relies on specialized techniques such as AC-STEM and synchrotron-based XAS, which are not widely accessible. In addition, scaling the process is hindered by difficulties in preserving single-atom sites at high catalyst loadings and in demonstrating catalyst stability beyond 100 h of operation.

Cu–N–C SACs have shown significant potential in the electrochemical synthesis of urea from CO_2_ and NO_3_^−^. A study reported a Faradaic efficiency of up to 28% for urea synthesis using Cu–N–C catalysts. This performance is attributed to the Cu–N_4_ coordination structure of the catalyst, which enhances CO_2_ reduction, while its modified sites facilitate NO_3_^−^ reduction, thereby promoting effective C–N coupling.^40^

Similarly, Ni–N–C has proven effective for the activation of CO_2_ and NO_3_^−^ in urea synthesis. Hua et al. developed an efficient and scalable method for synthesizing Ni–N–C catalysts using guar gum, an abundant and low-cost natural polymer, as a template. The resulting Ni–N–C catalyst features a tri-coordinated Ni–N_3_ configuration, which enhances catalytic activity, and a hierarchical porous structure that facilitates mass and charge transport. This catalyst demonstrated stable operation for over 70 h in a membrane electrode assembly, achieving a total energy efficiency of 41.0%.^95^

Bimetallic catalysts have shown synergistic interactions that improve the efficiency of C–N coupling in the electrochemical production of urea from CO_2_ and nitrogen-containing sources. For example, Cu–Fe alloys combine the advantages of both metals, in which Cu facilitates CO_2_ reduction to ∗CO intermediates, while Fe promotes the reduction of NO_3_^−^ or N_2_, enabling effective C–N bond formation.^96^

Cu–Zn bimetallic systems improve the adsorption and conversion of CO_2_ and nitrogen species, with research showing that the integration of Cu and Zn oxides can reach a Faradaic efficiency of up to 22.27% for urea synthesis at −0.8 V vs. RHE.^97^

Metal oxides and hydroxides have emerged as effective catalysts for electrochemical urea synthesis, owing to their ability to stabilize reaction intermediates and facilitate the co-reduction of CO_2_ and nitrogen species. Titanium dioxide (TiO_2_) with oxygen vacancies has enhanced activation of both CO_2_ and NO_2_^−^, promoting efficient C–N coupling. Mao et al. have highlighted oxygen vacancy-rich indium-doped titanium dioxide (Vo-In-TiO_2_) for efficiently synthesizing urea by co-reducing NO_3_^−^ and CO_2_. The catalyst achieves a Faradaic efficiency of 9.06% and a urea yield of 759.8 μg mg^−1^ h^−1^ at −0.65 V vs. RHE. Its high performance is due to the synergistic effect of oxygen vacancies (Vo) and In^3+^ ions: Vo sites facilitate NO_3_^−^ reduction to ∗NH_2_, while In^3+^ enhances CO_2_ capture and promotes the coupling of ∗NH_2_ and ∗CO_2_ to form urea. This “relay catalysis” approach offers a promising strategy for designing efficient urea-synthesis catalysts.^41^ Cobalt-based oxides like Co_3_O_4_ have redox-active surfaces that are well-suited for NO_3_^−^ reduction. Studies show that Co_3_O_4_ demonstrates strong electrocatalytic activity in converting NO_3_^−^ to NH_3_, a crucial intermediate in synthesizing urea.^98^

#### Noble metal alloys and hybrids

Alloys and hybrid-based noble metal catalysts (Au, Pd, and Pt) also show great potential to catalyze the electrochemical urea synthesis at ambient conditions. These materials have been widely employed in various electrocatalytic applications, including CO_2_ reduction, NH_3_ synthesis, H_2_ evolution, and NO_3_^−^ reduction reactions, and have indicated excellent performances.^99^^,^^100^

Owing to their unique electronic properties for controlling the formation of the desirable intermediates through C–N coupling, along with high chemical stability and excellent electrocatalytic activity, these catalysts are considered highly promising for urea electrosynthesis.^18^^,^^101^ Recent advancements have demonstrated the efficacy of Pt catalysts in the electrocatalytic oxidative coupling of carbon monoxide (CO) with NH_3_ to synthesize urea under mild conditions. Xiong et al. developed a method for synthesizing urea through electrocatalytic oxidative coupling between CO and NH_3_ on Pt catalysts. They obtained an optimal selectivity of approximately 70% for urea, maintaining over 50% selectivity across a broad range of applied potentials, with an electrocatalytic C–N bond formation rate reaching 100 mmol h^−1^ g^−1^. Mechanistic studies suggest that the Pt-catalyzed reaction produces cyanate from CO and NH_3_, which then undergoes the Wöhler reaction to yield urea.^35^ Bimetallic alloys of noble metals, such as Au-Pd and Pt-Ru, are pivotal in catalysis and electrocatalysis due to their synergistic effects from tailored electronic and geometric interactions.^102^ For example, Au–Pd alloy demonstrates high efficiency in oxidation reactions by combining Pd reactivity with the ability of Au to stabilize intermediates.^103^ These alloys exhibit tunable selectivity based on their composition and structure, and their inherent corrosion resistance is further enhanced through alloying, making them highly durable in harsh environments.^104^ Furthermore, bimetallic alloys composed of Pd^15^ and Au^38^ with copper nanoparticles were precisely engineered to incorporate structural defects, thereby enhancing the density of electroactive sites.

#### Earth-abundant and non-noble metals

Although noble metals such as Pt, gold (Au), and silver (Ag) exhibit excellent catalytic activity, their high cost and scarcity have driven significant interest in more abundant, cost-effective non-noble metal catalysts. These include transition metal-based catalysts (such as iron (Fe), nickel (Ni), copper (Cu), cobalt (Co), and manganese (Mn)), along with their alloy catalysts (like Cu-Fe), which offer comparable efficiency, improved durability, and beneficial synergistic interactions.^105^ Additionally, metal-organic frameworks (MOFs) offer notable benefits over conventional catalysts due to their high surface area and tunable properties. Their porosity and surface area improve interactions between active sites and reactants, as well as facilitate efficient mass transport of ionic electrolytes, a feature lacking in traditional catalysts such as hydroxides, perovskites, and metal oxides. Moreover, the adaptable metal ions in MOFs enhance the performance and stability of electron-transfer reactions.^106^ Carbon-based materials, such as doped graphene, promote efficient charge transfer and represent promising non-noble options. While such catalysts are cost-effective, sustainable, and customizable, they still face challenges in achieving the activity and selectivity of noble metals, maintaining stability under reaction conditions, and clarifying their operational mechanisms. Ongoing research focused on optimizing these materials and understanding their mechanisms is crucial for advancing scalable, commercially viable, sustainable urea production. This research includes rational design, surface engineering, and innovative synthesis techniques to enhance the active sites, electronic structure, and morphology of the catalysts. By fine-tuning these elements, it becomes possible to develop more efficient and stable non-noble transition-metal-based catalysts for sustainable urea production, making them suitable for large-scale industrial applications.^106^ Geng et al. developed a high-efficiency dual-site electrocatalyst for ambient urea synthesis by synthesizing graphitic carbon encapsulated amorphous iron and iron oxide nanoparticles on carbon nanotubes (Fe(a)@CFe_3_O_4_/CNTs) using a liquid phase laser irradiation method.^107^ The resulting electrocatalyst achieved a notable urea yield of 1341.3 ± 112.6 μg h^−1^ mg_cat_^−1^ and a faradaic efficiency of 16.5 ± 6.1%, at a potential of −0.65 V (vs. RHE) in a 0.1 M KNO_3_ electrolyte under ambient conditions. *In situ* FTIR and NMR analyses indicated that the Fe(a)@C sites selectively reduce NO_3_^−^ to ∗NH_2_ intermediates while the Fe_3_O_4_ sites reduce CO_2_ to ∗CO intermediates. This dual-active-site synergy lowers the energy barrier for C–N coupling, enabling efficient urea formation. This study presents a sustainable approach to urea production by utilizing waste nitrogen and carbon sources using earth-abundant, non-noble metals as an electrocatalyst.

#### Covalent organic frameworks

Covalent organic frameworks (COFs) are a novel category of porous, crystalline materials composed of small organic building blocks connected through strong covalent bonds involving light elements such as H, B, C, N, and O.^108^ The unique characteristics of COFs, including high porosity, well-ordered crystalline networks, lightweight nature, excellent thermal stability, and high surface area, make them highly suitable candidates for C–N coupling reactions.^109^

Due to their high surface area, tunable porosity, and low density, COFs have emerged as promising materials for facilitating C–N coupling reactions, including urea synthesis.^110^ These properties enable COFs to serve as efficient catalysts or catalyst supports, thereby enhancing the accessibility of active sites and promoting effective molecular interactions.^111^ In urea synthesis, COFs can stabilize metal nanoparticles or active catalytic sites, which are crucial for activating nitrogenous and carbonyl compounds. Additionally, the thermal and chemical stability of COFs ensures robust performance under the harsh reaction conditions typically required for C–N bond formation, making them excellent platforms for sustainable, recyclable catalytic systems in urea production.^110^ Li et al. developed three-dimensional COFs (3D COFs) functionalized with benzene, pyrazine, and tetrazine cores to modulate catalytic performance through varying nitrogen content. The obtained results demonstrate a progressive improvement in electrical conductivity, light absorption, charge separation efficiency, and co-adsorption capacity for NH_3_ and CO_2_ from benzene-to pyrazine-to tetrazine-containing COFs. Among them, the tetrazine-based COF shows the most efficient photocatalytic performance for urea production, reaching a yield of 523 μmol g^−1^ h^−1^, 40 and 4 times greater than the benzene- and pyrazine-based COFs, respectively. This enhanced activity, attributed to the nitrogen-rich heterocyclic environment, as validated by *in situ* spectroscopic studies and DFT calculations, offers a promising route to sustainable urea synthesis via photocatalysis.^110^

#### P-block element catalysts

Catalysts containing p-block elements, such as B, In, O, and N, have recently shown promising performance for sustainable electrocatalytic urea production due to their intriguing physicochemical properties and intrinsic poor hydrogen adsorption.^112^ These elements are critical in stabilizing active sites, modulating electronic environments, and enabling novel reaction pathways. For example, boron (B) based catalysts such as boron nanosheets,^113^ boron carbides,^114^ boron nitride nanosheets,^115^ and boron phosphide nanoparticles^116^ possess inherent empty p orbitals and therefore demonstrate efficient N_2_ activation or protonation; thus, they could serve as a useful dopant to introduce active sites on the surface of other materials.^112^ P-block elements are often integrated into transition metal-based catalysts to enhance synergistic interactions. The strong interaction between P-block elements and N 2p orbitals results in excellent adsorption and activation of nitrogen sources. Chen et al. incorporated the p-block element bismuth (Bi) into the 1T-MoS_2_ phase to synthesize a Bi_2_S_3_/MoS_2_ composite. Combining the excellent conductivity of 1T-MoS_2_ and the strong N_2_ adsorption and activation capabilities of Bi, the catalyst achieved an NH_3_ yield rate of 54.64 μg h^−1^ mg^−1^ and a high Faradaic efficiency of 58.56%. This study highlights the synergistic interaction between p-block elements and transition-metal-based catalysts in promoting efficient NH_3_ synthesis via nitrogen reduction.^117^ Carbon-based materials, such as graphene and nitrogen-doped carbon nanosheets, are p-block elements that act as sustainable supports for Pd or Cu nanoparticles, promoting better dispersion and reusability. These p-block-derived supports enhance catalytic performance in reactions such as the Suzuki-Miyaura coupling and urea electrosynthesis.^118^

In addition, the integration of defect-engineered p-block-element-based catalysts represents a cutting-edge strategy to enhance catalytic performance in C–N coupling reactions.^119^ Oxygen-deficient InOOH structures are an example of defect engineering in metal oxide catalysts, where creating oxygen vacancies significantly boosts catalytic efficiency. These vacancies act as electron-rich sites that enhance the adsorption of reactants, such as CO_2_ and nitrogen compounds, thereby stabilizing crucial intermediates, such as ∗CONH_2_, which are necessary for C–N bond formation. Furthermore, oxygen vacancies modulate the local electronic environment, thereby lowering the energy requirements for vital steps such as CO_2_ activation and N–H bond coupling. Such defects also enhance charge transfer, a crucial process for electrochemical reactions, including urea synthesis and cross-coupling reactions. Notably, the oxygen-deficient surfaces of InOOH reflect a design approach also found in hybrid catalysts, such as Cu-In oxides. In both cases, oxygen vacancies play a crucial role by enabling the simultaneous activation of CO_2_ and NO_3_^−^, a critical step in the electrochemical synthesis of urea.^120^

Grain boundary-rich Bi_2_Se_3_ nanosheets are an exciting example of how structural defects can enhance catalyst performance. The defective edges and grain boundaries possess a high density of unsaturated active sites, facilitating the adsorption of reactants such as aryl halides and amines. Due to its two-dimensional architecture, these defects enhance electron mobility, thereby accelerating the redox cycles essential for electrocatalytic C–N coupling. Moreover, these defects modulate the local environment of Bi and Se atoms, enabling tunable selectivity and facilitating suitable reaction pathways toward desired products. Bi_2_Se_3_ is an excellent example of how p-block elements utilize their flexible oxidation states and structural adaptability to enable efficient catalysis, offering a sustainable alternative to noble metal-based systems.^121^

As an innovative strategy, researchers have focused on the rational combination of the above-mentioned electrocatalyst candidates to suppress the formation of unwanted intermediates and block associated mechanistic pathways. This approach also aims to control the electronic behavior at the electrode/electrolyte interface, facilitating C–N bond activation and increasing the electrocatalysis efficiency.^68^ More importantly, identifying the optimized ratios of transition metals, noble metals, and p-block elements components, particularly when single-atom engineering of the constructions is involved, is essential to conceptually designing an effective catalyst. Accordingly, systematic efforts should focus on developing cost-effective and scalable methods to rapidly produce highly efficient electrocatalysts from these material families, enabling large-scale urea production for industrial applications.

#### Challenges and opportunities in electrocatalyst design and sustainable applications

Multiple electrochemical charge-transfer processes involving H^+^ and e^–^ species make C–N coupling reactions highly competitive and complex. This competition increases the energy barrier for the overall process, ultimately lowering the yield of urea formation and reducing the Faradaic efficiency. The key challenge in this area is to achieve simultaneous reductions of carbon sources and nitrogenous compounds, thereby forming favorable intermediates that lead to the selective formation of urea.

To overcome these challenges and achieve optimal urea production efficiency, an effective electrocatalyst should meet several key criteria, including facilitating the strong adsorption and efficient activation of the reactants on its surface, enhancing the C–N coupling reaction between the desired intermediates and active sites, and lowering the energy barrier of the favorable reaction steps while suppressing competing side reactions.

On one hand, researchers should consider the intrinsic characteristics of the reactants, especially nitrogen-containing compounds, to reduce the energy costs and promote the reaction more effectively. For instance, the solubility of N_2_ in an electrolyte medium is lower than that of NO_2_^−^ and NO_3_^−^. Moreover, its activation is much more difficult due to the strong chemical bond between the two nitrogen atoms.

On the other hand, key technical parameters, such as overpotential, Faradaic efficiency, current density, electrode stability, and C/N selectivity, should be carefully monitored and optimized to accurately evaluate and improve the performance of urea electrosynthesis.^2^

Conventional electrocatalytic technologies utilize applied electric current and voltage to the electrode surface, precisely controlling the reaction kinetics and directing the selectivity of specific reactions at the electrode surface. These capabilities are often difficult to achieve through traditional thermal or homogeneous catalytic methods.^122^ This electrochemical control enables the fine-tuning of reaction pathways under milder conditions, activating inert molecules and offering greater specificity in product formation, particularly in complex multi-electron processes such as urea electrosynthesis.^79^

Additionally, these systems often exhibit reaction rates that favor the formation of higher-valued product distributions, which is a significant advantage when considering scale-up and practical applications.^123^ Utilizing electrical energy to power modern electrocatalytic systems addresses the key challenges associated with traditional reaction conditions.^124^ This strategy eliminates the need for high-temperature and pressure conditions to drive the reactions effectively while preventing the emission of hazardous gases such as CO and CO_2_, which can have an adverse impact on the environment.^125^ Beyond this, in processes such as urea electrosynthesis, gases such as CO and CO_2_, as well as nitrogenous species such as NO and NO_2_^−^, serve as key reactants in the electrochemical reactions.^57^

Therefore, large-scale urea electrosynthesis could significantly contribute to reducing the effects of global warming by utilizing atmospheric CO_2_, thereby helping to mitigate climate change.^126^ At the same time, it can contribute to air purification in urban areas by converting toxic gaseous pollutants, such as CO and NO, commonly emitted by industrial activities and vehicles, into valuable chemical products.^127^

Conventional catalytic methods are limited by steady reaction conditions and energy-intensive processes, whereas electrosynthesis offers a high degree of tunability, enabling precise control over product purity, sustainability, and selectivity.^128^ This adaptability makes electrosynthesis approaches particularly well-suited to meet the goals of green chemistry and sustainability, offering cleaner, more efficient, and environmentally friendly alternatives to traditional processes.^129^^,^^130^ However, a significant challenge in electrocatalysis lies in designing and constructing highly stable electroactive surfaces that can function effectively as catalysts to ensure long-term stability under reaction conditions while maintaining high activity and selectivity in practical electrosynthesis applications.^131^ Utilizing renewable materials to achieve carbon neutrality and promote long-term sustainability has emerged as a highly promising strategy, garnering significant attention over the past few years.^132^ However, the high energy requirements in manufacturing single-atom engineered catalysts^133^ and the lack of a precise mechanistic illustration of the interfacial phenomena^134^ limit their practical applications. Within these concepts, the scientific community should significantly improve the fundamentals of electrosynthesis by establishing robust standards, conducting detailed product analyses, evaluating the long-term stability of electrode architectures, and developing large-scale electrochemical systems.^135^ Another significant challenge in industrial electrocatalysis is the demand for high-concentration electrolytes and high-temperature electrolysis conditions to achieve efficient operation.^132^

However, elevated temperatures often compromise the stability of most electroactive materials, particularly at the laboratory scale. This issue is further worsened by using high-concentration electrolytes, which create highly corrosive environments for testing. Overcoming these challenges is essential to establishing synthetic electrocatalysis as a robust and scalable tool that facilitates its transition from laboratory research to real-world applications.

## Photocatalytic synthesis of urea

Due to their notable safety, eco-friendly nature, and simplicity of operation, solar-driven photocatalytic urea synthesis systems have recently gained considerable attention as a sustainable and adaptable alternative to the Bosch–Meiser process.^136^^,^^137^ Photocatalysis involves the generation of multiple electron-hole pairs upon absorbing light photons on the surface of solid materials.^138^ The inherent potential of photocatalytic materials can be improved by creating electron-rich sites in their crystal lattice. These sites are created through strategies such as defect, interfacial, facet, and junction engineering, as well as heteroatom doping and precise control over structural morphology.^139^ Achieving efficient electron transfer to drive the feasible multi-electron reduction of nitrogenous reactants (NO, N_2_, NO_3_^−^, NO_2_^−^, NH_3_) and CO_2_ during C–N coupling reactions is crucial but challenging for urea photosynthesis.

The earliest report on urea photocatalytic synthesis was published in 1998 by Kuwabata et al.^140^ Simultaneous reduction of CO_2_ and NO_3_^−^ ions occurred on the surface of nano-sized TiO_2_ supported on a polyvinylpyrrolidone gel film (TiO_2_/PVP). This group achieved a urea yield of 1.9 mM after 5 h of reaction time under irradiation from a 500 W high–pressure mercury arc lamp. Interestingly, the systematic investigation of the reaction pathway revealed that the reduction of NO_3_^−^ ions is the rate-limiting step in urea production over the photocatalysts. Subsequently, a variety of titanium oxide-based photocatalysts, such as Cu-containing TiO_2_ particles,^141^ polymeric TiO_2_ composites,^142^ and zeolite-based Fe-TiO_2_ structures,^136^ have been successfully developed for urea synthesis from different reactants. Over the last decade, significant research efforts have been focused on designing advanced heterojunction architectures and supporting photocatalyst systems to enhance urea production yields and understand surface reaction mechanisms.

**Figure 5:**
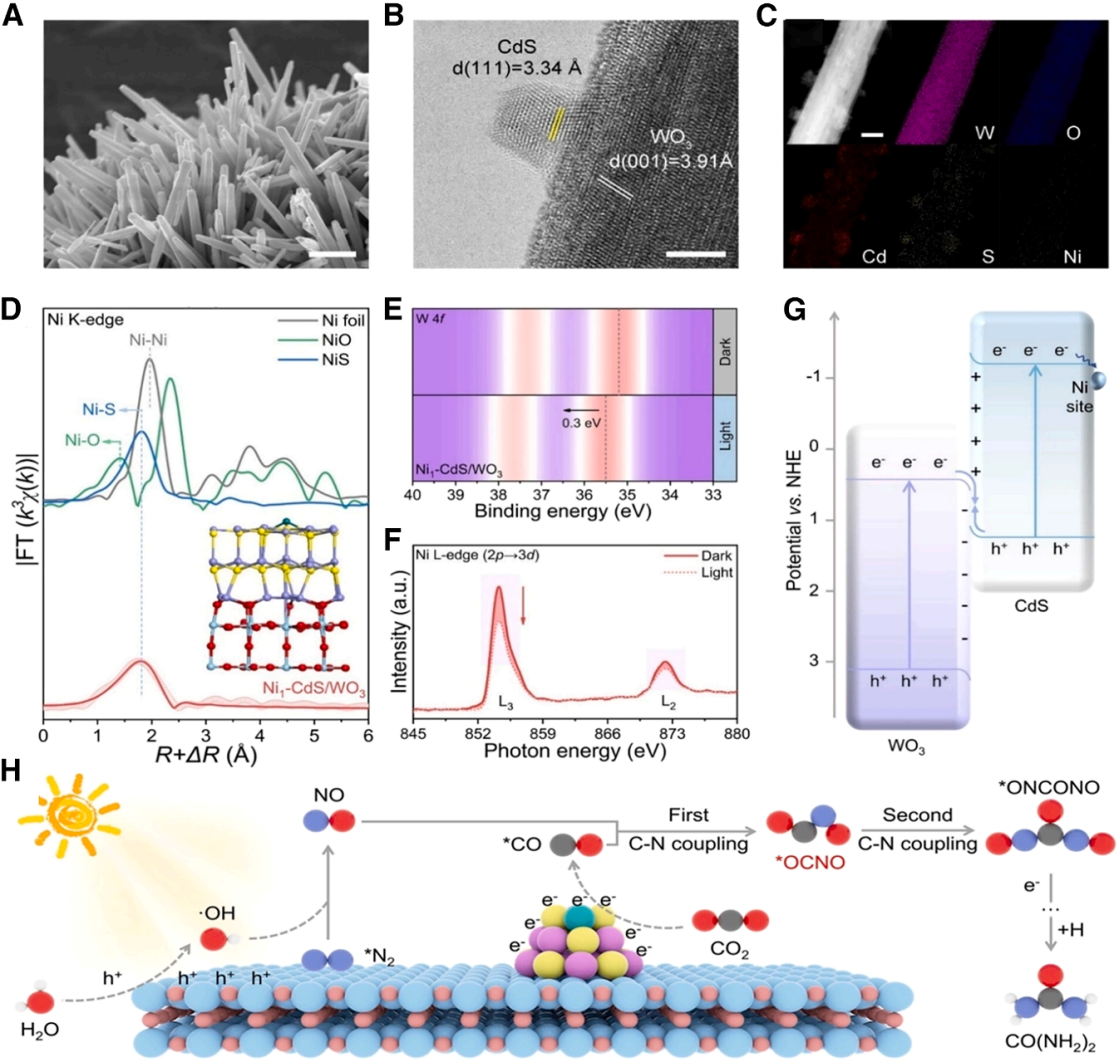
The WO_3_ and Ni_1_-CdS/WO_3_ nanostructures were synthesized, and their structural characterization and the mechanism of photogenerated carrier in them are demonstrated (A) SEM image of WO_3_ nanorods (scale bars, 500 nm). (B) HRTEM image of Ni_1_-CdS/WO_3_ (scale bars, 5 nm). (C) Elemental mapping images of Ni_1_-CdS/WO_3_ (scale bars, 50 nm). (D) The Ni K-edge spectra of Ni1-CdS/WO_3_ in R space, (inset: corresponding geometric configuration for Ni_1_-CdS/WO_3_, in which the green = Ni atom, mauve = Cd atom, yellow = S atom, red = O atom, and indigo = W atom. (E) *In situ* XPS tests in W 4f spectra over Ni_1_-CdS/WO_3_. (F) *In situ* Ni L-edge tests of Ni_1_-CdS/WO_3_. (G) Flow direction of photogenerated carriers in Ni_1_-CdS/WO_3_. (H) Photocatalytic urea synthesis mechanism over Ni_1_-CdS/WO_3_ catalyst.^143^ Copyright@2025, Wiley Online Library.

For instance, Zhang et al. utilized a Ni_1_-CdS/WO_3_ heterojunction as an efficient photocatalytic approach for urea synthesis from CO_2_ and N_2_.^143^ The catalyst achieved a urea yield rate of 78 μMh^−1^, an apparent quantum yield of 0.15% at 385 nm, and a solar-to-urea conversion efficiency of 0.024%. Mechanistic studies reveal that this catalyst effectively uses photogenerated electrons and holes to convert CO_2_ and N_2_ to urea. The CO_2_ adsorbed on the Ni site was reduced into ∗CO by photogenerated electrons. While the photogenerated holes reacted with H_2_O to generate ^·^OH radicals, further converting the N_2_ adsorbed on the WO_3_ surface into NO species. As shown in Figure 5A, the scanning electron microscopy (SEM) image of WO_3_ nanorods displayed an average diameter of ∼100 nm with smooth surfaces. The high-resolution transmission electron microscopy (HRTEM) image of Ni_1_-CdS/WO_3_ showed two distinct sets of fringes with interplanar spacings of 3.34 and 3.91 Å, which are attributed to the (111) crystal plane of CdS and the (001) crystal plane of WO_3_, respectively. (Figure 5B). Additionally, the elemental mapping images of Ni_1_-CdS/WO_3_ reveal a uniform distribution of Ni, Cd, S, W, and O elements (Figure 5C). Ni K-edge X-ray absorption fine structure (XAFS) was used to further examine the chemical states and coordination structures of the Ni species. The Ni K-edge spectra in R space, shown in Figure 5D, reveal a prominent peak at approximately 1.8 Å, attributed to the Ni-S coordination. No distinct peaks for Ni–O or Ni–Ni bonds were detected, confirming the atomic dispersion of Ni species on the CdS surface in Ni_1_-CdS/WO_3_. Additionally, *in situ* X-ray photoelectron spectroscopy (XPS) measurements were performed to investigate the direction of photogenerated carrier flow in the heterojunctions. As demonstrated in Figure 5E, under illumination, the CdS/WO_3_ showed a 0.2 eV positive shift in the W 4f spectra and a 0.4 eV negative shift in the Cd 3d spectra compared to the dark conditions, which indicates that the photogenerated electrons and holes are primarily concentrated in the CdS and WO_3_ components, respectively. A similar trend was observed for Ni_1_-CdS/WO_3_, except that the photogenerated electrons, which initially accumulated at CdS, were further transferred to the Ni single atoms. The *in situ* Ni L-edge X-ray absorption spectra of Ni_1_-CdS/WO_3_ under different conditions are shown in Figure 5F, confirming electron transfer to the Ni sites. Compared to the dark condition, the Ni L-edge peak intensity decreased significantly under illumination, attributed to photogenerated electrons occupying the unoccupied Ni 3d orbital. The schematic illustration of the flow direction of photogenerated carriers in Ni_1_-CdS/WO_3_ is shown in Figure 5G. The differences in work functions between CdS and WO_3_ triggered electron transfer when they contacted each other, generating an internal electric field at their interface. This field caused electrons to move from CdS to WO_3_, leading to band bending in both materials. As a result, the photogenerated electrons in the conduction band (CB) of WO_3_ were driven toward CdS, where they recombined with the photogenerated holes in the valence band (VB) of CdS. At the same time, electrons in the CB of CdS and holes in the VB of WO_3_ remained separated, promoting the reduction and oxidation reactions. The introduction of Ni single atoms further facilitated the transfer of photogenerated electrons from CdS to the Ni sites, improving the separation of the photogenerated carriers in Ni_1_-CdS/WO_3_. Figure 5H presents the schematic illustration of the photocatalytic urea synthesis mechanism over the Ni_1_-CdS/WO_3_ catalyst. When illuminated, photogenerated electrons and holes accumulate on the Ni_1_-CdS and WO_3_ components, respectively. CO_2_ is first adsorbed on the Ni site, where it is reduced to ∗CO by photogenerated electrons. At the same time, photogenerated holes react with H_2_O to generate ^·^OH radicals, which then convert N_2_ adsorbed on the WO_3_ surface into NO species. These NO species move to the Ni site, where they react with ∗CO in a series of steps to form ∗OCNO and ∗ONCONO intermediates. Finally, ONCONO undergoes hydrogenation via a multi-step proton-electron-coupling process, yielding urea.

Two heterojunction composites, S-defect engineered NiCoP/ZnIn_2_S_4−x_^144^ and D-CdS@3D-BiOBr,^145^ have been successfully examined for visible-light-driven photocatalytic urea synthesis. The interfacial charge transfer at the semiconducting junctions effectively maintained a high concentration of photogenerated electrons, thereby enhancing catalytic performance. Based on mechanistic understanding, the photocatalytic activity of these structures is further examined in the following sections.

**Figure 6:**
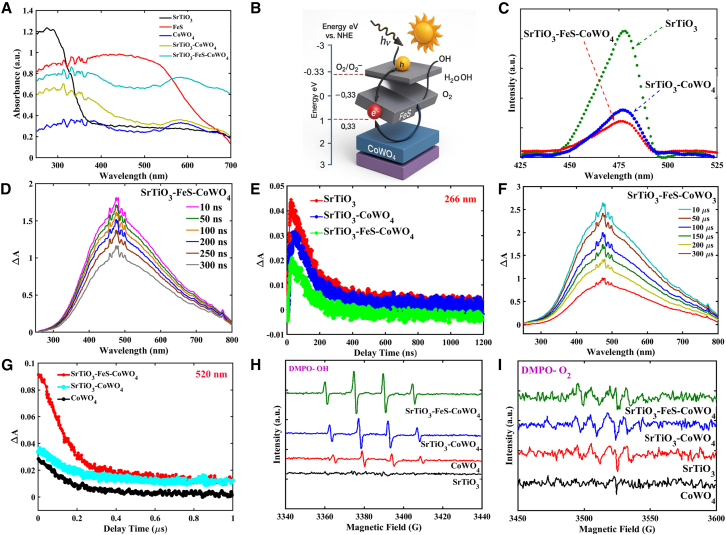
Optical characterization (A) The results of DRS. (B) Heterojunction’s energy band diagram of the construction. (C) PL spectra. (D and E) Transient absorbance spectra (on nanosecond timescale) of the ternary photocatalyst, and the synchronized kinetic trace plots at 266 nm. (F and G) The results of transient absorbance spectroscopy (on a microsecond timescale) of the final construction, and the analogous kinetic trace plots at 520 nm, the results of EPR for detecting: (H) hydroxyl radicals and (I) superoxide ion-radicals. Adapted with permission from ref.^146^ Copyright 2024, Wiley Online Library.

A novel Z-scheme heterojunction, SrTiO_3_-FeS-CoWO_4_, has been recently examined for photocatalytic urea generation from CO_2_ and N_2_.^146^ An excellent urea production yield of 8054.2 μg ⋅ g_cat_^−1^ ⋅ h^−1^ indicates that the performance of the fabricated system is significantly higher than the current state-of-the-art technologies. The enhanced charge transfer facilitated by the FeS semiconducting bridge during construction helps preserve the photogenerated electrons, enabling the efficient co-reduction of the precursors and promoting the progress of C–O coupling. Furthermore, the engineered photocatalysts benefit from three distinct types of metal active sites, each selectively promoting the formation of desired products while minimizing impurity concentrations. Diffuse reflectance spectroscopy (DRS) results revealed that the ternary system exhibited significantly enhanced visible-light absorption compared to its pure and dual components (Figure 6A), partly due to surface plasmon resonance effects of iron sulfide. Mott–Schottky plots were employed to determine the flat band potentials and Fermi levels, which subsequently enabled the calculation of the conduction band (CB) and valence band (VB) positions for constructing the energy band diagram of SrTiO_3_-FeS-CoWO_4_ (Figure 6B). Furthermore, ultrafast spectroscopic analysis (Figures 6C–6E) confirmed the Z-scheme charge-transfer mechanism, as evidenced by both photoluminescence (PL) and transient absorption spectroscopy (TAS), demonstrating highly efficient interfacial charge transfer at the SrTiO_3_-FeS-CoWO_4_ junctions, thereby significantly enhancing photocatalytic performance. Transient absorbance spectroscopy (Figures 6F and 6G) revealed that the synergistic integration of the three semiconducting components significantly extends the average lifetime of the electron-hole pairs through efficient charge separation. This improvement is attributed to accelerated charge migration and suppressed recombination, enabling electrons accumulated in the system to simultaneously reduce CO_2_ and N_2_ effectively.

Meanwhile, the photogenerated holes facilitate H_2_O oxidation to produce H^+^, thereby supporting the reduction reactions and preventing the oxidation of N_2_. Additionally, electron paramagnetic resonance (EPR) analysis was employed further to elucidate the charge-transfer mechanism within the constructed system. The results indicated a substantial generation of hydroxyl radicals and a relatively weak signal for superoxide ion radicals in the system. This observation can be readily explained by the band edge potentials of the individual semiconducting components (Figures 6H and 6I).

### Reaction pathways in photocatalytic urea production

In general, heterogeneous photocatalysis involves illuminating a solid semiconductor, resulting in the rapid generation of electron-hole pairs that produce reactive radicals that drive oxidation-reduction reactions.^147^ Using instrumental techniques such as electron spin resonance (ESR) spectroscopy^148^ and analyzing band electrochemical potentials, as well as experimental methods such as scavenging tests,^149^ researchers were able to describe the charge migration trends and identify the primary reactive species involved in photocatalysis. Subsequently, benefiting from the *in situ* identification of reaction intermediates and the previous findings, a well-supported mechanistic illustration of the process could be presented. Due to the extensive research on charge transfer mechanisms at the photocatalyst interface, this section focuses on the reaction mechanism of urea formation, with an emphasis on intermediate identification.

According to Kuwabata et al.,^140^ photocatalytic urea formation involves simultaneous reduction reactions of the reactants, in this case, NO_3_^−^ and CO_2_. Accordingly, the overall process requires 16 electrons to be transferred through a multi-electron reaction as follows:

(Equation 22) $${2\text{NO}}_{3}^{-}+{\text{CO}}_{2}+{18H}^{+}+{16e}^{-}\to {\text{NH}}_{2}\text{CO}{\text{NH}}_{2}+7H_{2}O$$

The formation of urea under photocatalytic conditions may proceed via additional reaction pathways (Equations 23, 24, 25, and 26), depending on the nature of the precursors involved^136^:

(Equation 23) $${2\text{NO}}_{3}^{-}+\text{CO}+{16H}^{+}+{14e}^{-}\to {\text{NH}}_{2}\text{CO}{\text{NH}}_{2}+6H_{2}O$$

(Equation 24) $${2\text{NO}}_{3}^{-}+\text{HCOOH}+{16H}^{+}+{14e}^{-}\to {\text{NH}}_{2}\text{CO}{\text{NH}}_{2}+7H_{2}O$$

(Equation 25) $$2{\text{NH}}_{2}\text{OH}+{\text{CO}}_{2}+{4H}^{+}+{4e}^{-}\to {\text{NH}}_{2}\text{CO}{\text{NH}}_{2}+3H_{2}O$$

(Equation 26) $$2\text{NO}+{\text{CO}}_{2}+{10H}^{+}+{10e}^{-}\to {\text{NH}}_{2}\text{CO}{\text{NH}}_{2}+3H_{2}O$$

All the above-mentioned reactions proceed through multi-electron steps where the required electrons can be supplied by photocatalysis activation and electron-rich sites within the semiconductor crystal structures. For instance, Mohamed et al. suggested a multi-step reaction mechanism (Equations 27, 28, 29, and 30) describing the reduction of NO_3_^−^ ions on TiO_2_ nanoparticles^150^:

(Equation 27) $${\text{NO}}_{3}^{-}+{2H}^{+}+{2e}^{-}\to {\text{NO}}_{2}^{-}+H_{2}O$$

(Equation 28) $${\text{NO}}_{2}^{-}+{2H}^{+}+{2e}^{-}\to {\text{NO}}^{-}+H_{2}O$$

(Equation 29) $${\text{NO}}^{-}+{3H}^{+}+{2e}^{-}\to {\text{NH}}_{2}\text{OH}$$

(Equation 30) $${\text{NH}}_{2}\text{OH}+{3H}^{+}+{2e}^{-}\to H_{2}O+{\text{NH}}_{4}^{+}$$

According to Srinivas et al., the photogenerated NH_3_ can react with CO_2_ on the surface of the supported TiO_2_ photocatalysts via another multi-electron mechanism to produce urea^151^:

(Equation 31) $${\text{NH}}_{4}^{+}+{\text{CO}}_{2}+e^{-}\to {\text{NH}}_{2}\text{COO}{\text{NH}}_{4}$$

(Equation 32) $${\text{NH}}_{2}\text{COO}{\text{NH}}_{4}\;\to H_{2}O+{\text{NH}}_{2}\text{CO}{\text{NH}}_{2}$$

**Figure 7:**
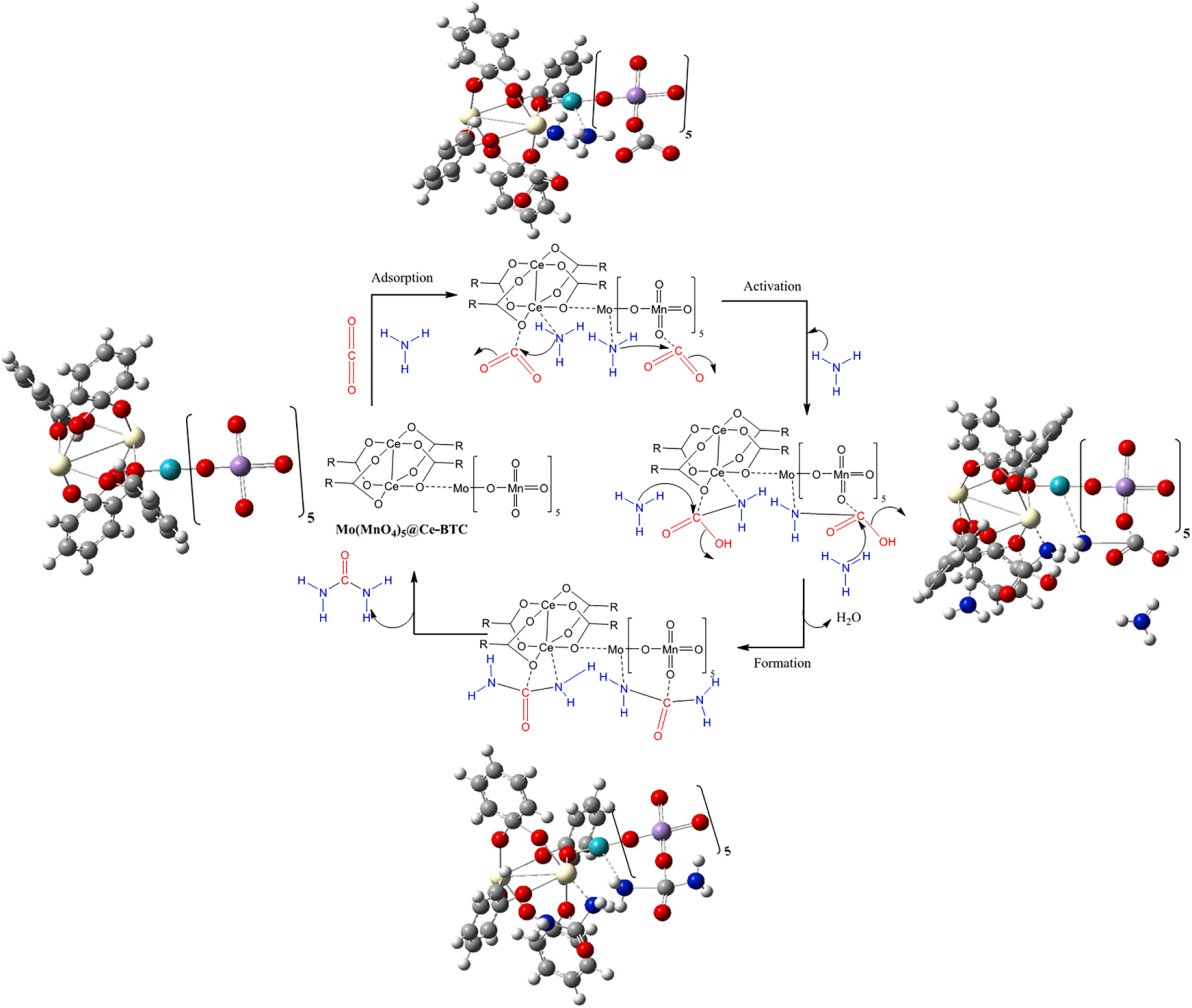
Mechanistic illustration of urea formation on the surface of Mo(MnO_4_)_5_@Ce-BTC photocatalyst, considering the metal active sites’ tendency to better promote the multi-electron reaction pathway Adapted with permission from ref.^137^ Copyright 2024, Nature.

Based on the mechanisms presented in the preceding equations, heteroatom-metal active sites could play a distinctive role in directing the selective formation of urea on the surface of MOF-based photocatalysts, as reported by El-Shahat and Abdelhameed.^137^ They provided a detailed description of the electron-hole migration pathways across the junctions of the Mo(MnO_4_)_5_@Ce-BTC photocatalyst, demonstrating its ability to generate a sufficient population of photogenerated electrons to drive the simultaneous reduction reactions of CO_2_ and NH_3_. The mechanism of urea generation, involving the utilization of photogenerated electrons and active sites of Mo, Mn, and Ce on the catalyst surface, is proposed as shown in Figure 7. Initially, CO_2_ and NH_3_ molecules are adsorbed onto the active sites of the catalyst, including Ce sites in the Ce-BTC framework and Mo–Mn oxide sites from the doped nanoparticles. Upon exposure to visible light, electrons are excited from the VB to the CB of two metal compounds, leaving behind holes in the VB. Photogenerated electrons then migrate from the CB of Ce-BTC to the CB of Mo–Mn, while the holes move from the VB of Ce-BTC to the VB of Mo–Mn. The heterojunction between Ce-BTC and Mo(MnO_4_)_5_ promotes efficient separation of photogenerated electrons and holes, reducing recombination. During the reaction, electrons take part in the coupling mechanism. It is suggested that the photocatalytic mechanism for urea generation involves the coupling of NH_3_ with adsorbed CO_2_ on the Mo(MnO_4_)_5_@Ce-BTC composite, forming the intermediate H_2_NCOOH. This intermediate then reacts with NH_3_ to produce ammonium carbamate (H_2_NCO_2_NH_4_), which undergoes a one-step hydrogenation to form urea through a multi-step process, as outlined in the reaction pathways of Equations 31 and 32.

Liang et al. investigated urea synthesis using noble-metal-free NiCoP/ZnIn_2_S_4−x_ photocatalysts under ambient conditions and proposed that the reduction of CO_2_ to CO is the first step in the mechanism. Photo-excited ∗N=N∗ species reacted with CO to form ∗NCON∗, the primary intermediate in urea formation, which is then hydrogenated to produce urea.^144^ Spin-polarized electrons generated by the bi-functional photocatalyst were demonstrated as the primary driving force for urea production in this system. In another study, Maimaiti et al. investigated the synergetic interaction between non-metal defects, specifically oxygen vacancies and metal (Ti) active sites on the surface of Ti^3+^-doped TiO_2_ nanoparticles supported on the carbon nanotubes with iron cores (Ti^3+^-TiO_2_/Fe-CNTs) as a photocatalyst.^152^ According to their report, the dual catalytic active sites, oxygen vacancies, and Ti^3+^ jointly facilitate the reduction of CO_2_ and N_2_ for urea synthesis through an optimal spatial arrangement. Ti^3+^ species specifically enhance the adsorption of N_2_, which is subsequently activated by the photogenerated electrons to form intermediates such as –NH–NH–, NH_2_–, and –N=N–. Simultaneously, CO_2_ molecules are adsorbed at the oxygen vacancy sites located near Ti^3+^ centers, where they undergo a structural transformation from the linear to the geniculate configuration. Subsequently, the C–O bonds are broken, facilitating C–N coupling and ultimately leading to the formation of urea.

In a related study, Xia et al. investigated the photocatalytic activity of a series of Pd-decorated molecular sieve LTA-3A (referred to as Pd-LTA-3A photocatalysts) for solar-driven urea synthesis.^153^ Acting as the main photothermal driving force of the urea formation reaction, Pd nanoparticles catalyzed the NH_3_ dissociation on the catalyst surface. Meanwhile, the zeolite support played a complementary role by adsorbing the H_2_O byproduct, thereby shifting the reaction equilibrium toward increased urea production. A mechanistic illustration of the reaction, supported by the diffuse reflectance infrared Fourier transform spectroscopy (DRIFTS) and DFT calculations, revealed that Pd–NH_2_ and Pd–H are the most likely nitrogen-containing intermediates formed immediately after the NH_3_ dissociation at the surface-active sites. These intermediates can rapidly form carbamate intermediate species through nucleophilic addition to CO_2_. The subsequent intermediates, NHCO and NHCONH_2,_ are generated via an oxidation step followed by a second nucleophilic addition reaction involving Pd–NH_2_, with Pd–H contributing. These intermediates then react with Pd–H, ultimately leading to the formation of urea.

Irfan et al.^146^ proposed a direct reduction pathway of N_2_ and CO_2_ leading to urea formation (Equation 33), based on the emergence of the prominent peaks attributing to the stretching vibrations of NH_2_ (3623 cm^−1^) and NH (3702 cm^−1^) intermediates, along with the characteristic peak at 1440 cm^−1^ corresponding to C–N bonding.

(Equation 33) $$N_{2}+{\text{CO}}_{2}+{6H}^{+}+{6e}^{-}\to {\text{NH}}_{2}\text{CO}{\text{NH}}_{2}+H_{2}O$$

To further investigate the urea formation pathway on the SrTiO_3_-FeS-CoWO_4_ photocatalyst surface, a systematic DFT calculation was conducted. Based on the results, the following energetically favorable route was proposed for urea generation under the photocatalytic condition (Equations 34, 35, 36, 37, 38, and 39)^146^:

(Equation 34) N2+CO2→N2∗+CO2∗

(Equation 35) N2∗+CO2∗→N2∗+COOH∗

(Equation 36) N2∗+COOH∗→N2∗+CO∗

(Equation 37) N2∗+CO∗→NCON∗

(Equation 38) NCON∗→NCHONH∗

(Equation 39) NHCONH∗→NH2CONH2

The C–N coupling was identified as the rate-determining step; however, due to the presence of dual metallic active sites that facilitate the adsorption and activation of N_2_ and CO_2_, the SrTiO_3_-FeS-CoWO_4_ photocatalyst demonstrated a significantly enhanced ability to lower the energy barriers associated with this crucial step, compared to the single and dual semiconducting components.

Based on the above statements, the development of environmentally friendly approaches, such as photocatalysis, for the clean and efficient production of high-value-added urea is gaining increasing attention among researchers. Due to rapid advances in emerging instrumental techniques for mechanistic elucidation, further advancements in the rational design and scaling-up of reactors and photoreaction systems are expected in the near future.

### Challenges in photocatalytic urea synthesis from a catalyst perspective: Side reactions, selectivity, and yield

Urea synthesis requires simultaneous reduction of both carbon and nitrogen precursors. Therefore, photogenerated electrons are the vital driving force for the reduction steps in light-driven urea production systems.^154^ However, positively charged holes with strong oxidizing capability threaten the efficiency and selectivity of the system by adversely interfering with the simultaneous reduction reactions of the carbonous and nitrogenous sources.^155^ When NO_3_^−^ is used as the nitrogen precursor in aqueous photocatalytic urea synthesis, side reactions significantly impact selectivity and overall yield. In photocatalytic NO_3_^−^ reduction systems, NO_3_^−^ rarely forms a single product; instead, it produces multiple competing products, including NO_2_^−^, NH_4_^+^, and N_2_, with a selectivity strongly dependent on the catalyst type and reaction conditions. In the presence of H_2_O, photogenerated holes can oxidize H_2_O molecules and generate reactive ^·^OH radicals, which could facilitate the cyclic reductant reformation of NO_3_^-^.^156^ These side products form because photogenerated holes and ^·^OH radicals not only consume charge carriers that would otherwise accelerate reductive C–N coupling but also re-oxidize reduced nitrogen intermediates back toward NO_3_^−^ or other less reduced states, decreasing the effectiveness of NO_3_^−^ utilization. As a result, urea selectivity in NO_3_^−^-based systems is fundamentally limited by competing oxidative pathways that divert electrons and holes toward byproduct formation, highlighting the need for catalyst designs that suppress these side reactions while promoting selective C–N bond formation.^156^ Conversely, ^·^OH non-selectively oxidizes organic intermediates, essential for urea synthesis,^136^ and can degrade the urea produced. Park et al. demonstrated that a large amount of photogenerated holes and highly reactive ^·^OH radicals are produced from H_2_O oxidation on the TiO_2_ surface under UV irradiation, which could readily drive non-selective oxidation reactions.^157^ Nonselective reactions become problematic for urea synthesis because the same holes and ^·^OH radicals compete with the desired reductive C–N coupling, consuming charge carriers and oxidizing key intermediate, effectively lowering the net selectivity toward urea and diverting reactive electrons and holes into oxidative pathways. Together, these findings illustrate that photogenerated holes and ^·^OH species act as major sinks for reactive electrons and holes, reducing selectivity and effective urea yield unless their formation and reactivity are carefully controlled through catalyst design or operating conditions. Experimental explorations of the photocatalytic NO_3_^−^/CO_2_ co-reduction and photoelectrochemical urea synthesis indicated that under ambient conditions, some undesired byproducts such as NO_2_^−^, NH_4_^+^, and even H_2_O_2_ could be formed alongside urea, reflecting the poor selectivity toward strategic goals. For example, photoelectrochemical reduction of NO_3_^−^ with CO_2_ over a Cu_2_O photocathode produced urea at a rate of about 29.7 μmol g^−1^ h^−1^ with a relatively low Faradaic efficiency of ∼12.9%, indicating that nearly 90% of the electrons are consumed by side reactions rather than contributing to urea formation.^158^ Another harmful consequence of ^·^OH activity is the dimerization of N_2_O to toxic N_2_O_4_, exposure to 100 ppm of which can cause pulmonary edema or even be fatal.^159^^,^^160^ Under the applied irradiation conditions, particularly with UV light, direct photolysis of the reactants may occur as all possess unsaturated chemical bonds in their molecular structures. The work by Wang et al. shows that photocatalytic urea synthesis using CO_2_, N_2_, and H_2_O faces the typical selectivity challenges inherent to aqueous photocatalytic C–N coupling.^161^ In the visible-light-driven photocatalytic system employing CdS@Bi_2_WO_6_ for the co-reduction of CO_2_ and N_2_ in H_2_O, the reported reaction yield of urea is modest but notable, with the optimized 20 wt % CdS@Bi_2_WO_6_ catalyst achieving a production rate of approximately 10.0 μmol g^−1^ h^−1^ under visible illumination. The existence of competing side reactions, such as the hydrogen evolution reaction (HER), the partial reduction of CO_2_ to carbonaceous intermediates such as CO or HCOO^−^, and the N_2_ reduction to NH_3_ or other nitrogenous byproducts, is common in systems of this type. These side pathways divert charge carriers away from the targeted C–N coupling and decrease the efficiency of urea formation, highlighting the ongoing need for catalyst designs that enhance charge separation, suppress non-selective reduction and oxidation reactions, and steer electrons specifically toward urea production. Other research has reported that NO_3_^−^ photolysis under ultraviolet irradiation in the 270–330 nm wavelength range generates ^·^OH radicals, consumes some reactants, and thereby shifts the equilibrium of photocatalytic reactions toward a lower photocatalytic yield.^162^ The findings from this research on NO_3_^−^ photodissociation, particularly via spin-forbidden singlet–triplet transitions, could be applied to broader photochemical processes, such as urea synthesis, particularly in the design of productive systems for light-driven reactions and catalysis. Specifically, understanding how excited triplet states influence reactions could be important for optimizing light-driven urea-forming processes in artificial photosynthesis or solar-based chemical synthesis. Similar reactions could be expected for NO, NO_2_^−^, and N_2_ precursors. Recent theoretical studies have indicated that direct photolytic dissociation of CO_2_ into CO + O through singlet and triplet pathways can occur in the range of 150–210 nm and provides molecular-level photodissociation dynamics and cross-sections.^163^ This suggests that if such dissociation occurs during CO_2_ reduction processes, it may interfere with the desired C-O coupling reactions, potentially leading to less efficient or undesirable product formation. Another challenge in the field is the generation of superoxide radical ions (^·^O_2_^−^) through the consumption of photogenerated electrons by molecular oxygen.^164^ Although ^·^O_2_^−^ has sufficient potential to produce environmentally friendly O_2_ and regenerate nitrogenous precursors such as NO_2_^−^ by reacting with other photoexcited species in the reaction medium,^165^ a significant concern is the strong affinity of urea to attach this species,^166^ as demonstrated by both theoretical and experimental studies. As mentioned earlier, photooxidation and other undesired photoreduction pathways are unavoidable in photocatalytic systems. These side reactions can significantly influence reaction selectivity and pose challenges for scaling up systems for real-world applications. Like other photocatalysis-based technologies, there are considerable scalability challenges, among which the stability of photocatalysts and their anti-corrosion properties, mass transfer issues, and reactor design complexities need to be substantially addressed.^167^^,^^168^ As a technical point, the adsorption capacity of reactants on the surface of photocatalysts, closely linked to surface texture engineering, is critical for reactant activation and overall efficiency and is considered a key stage in photocatalyst design and fabrication.^169^ For instance, N_2_, as a nitrogenous source of the urea photocatalytic reaction, is almost inert and should be sufficiently activated to participate in the C–O coupling photoreactions.^170^ Single-atom engineering at the surface and interface of materials leverages the optoelectronic properties of unique heteroatoms to enhance the adsorption and activation of carbon and nitrogen precursors, providing a rational strategy to overcome these challenges.^171^^,^^172^ Adsorption/activation challenges indeed affect the scalability of the other two catalytic urea synthesis approaches, including electrocatalysis^173^ and photoelectrocatalysis,^174^ as well as those not limited to photocatalysis. Optimizing the large-scale design of photoreactors and the physicochemical properties of catalysts poses significant economic challenges for the development of photocatalysis technologies.^175^

Additionally, employing reliable and durable illumination sources for large-scale photocatalytic systems increases energy costs, making them less competitive with conventional urea synthesis methods.^176^ Integrating photocatalytic and electrocatalytic approaches offers a promising solution to many of the technical challenges associated with each technique, aiming to develop practical constructions for the large-scale production of high-quality urea. This idea will be comprehensively discussed in detail in the following section.

Based on the statements discussed above, photocatalysis has attracted growing attention in recent years as a sustainable approach.^26^ Although the large-scale implementation of this approach at the pilot plant level raises cost concerns, limiting its economic viability, photocatalysis offers distinct environmental advantages and benefits from significant advances in material engineering at the atomic and molecular levels. The coexistence of photo-oxidation pathways, such as H_2_O oxidation to ^·^OH radicals and reduction reactions (CO_2_/N_2_ activation), in photocatalytic urea synthesis systems poses a fundamental challenge, as both processes compete for photogenerated electrons and holes. This competition directly reduces urea production yield and selectivity by diverting charge carriers toward side reactions instead of C–N coupling.^146^^,^^177^

Moreover, studies have reported on the direct photooxidation of urea through radical-mediated reactions under irradiation.^157^^,^^178^ Accordingly, appropriate strategies should be implemented to minimize the undesirable impacts of photogenerated holes in these systems. The addition of external hole scavenging agents, often used to reduce hole concentration in photocatalysis, may not be an appropriate approach as these substances are not easily separable and can, hence, interfere with final product purity. Despite the limited number of studies on the photocatalytic synthesis of urea in recent years (Table 3), the existing drawbacks of photocatalytic synthesis systems should be prioritized rather than solely advancing the design of new photocatalytic materials.

**Table 3:** A brief overview of the recent photocatalytic systems developed for urea production over the past few years

Photocatalyst	Reactants	Electrolyte composition	Irradiation source, light intensity	Reaction rate	Reference
NiCoP/ZnIn_2_S_4−x_	N_2_ + CO_2_	H_2_O	visible light (>420 nm), 100 mW/cm^2^	19.6 μmol g^−1^ h^−1^	Liang et al.^144^
Pd/LTA-3A	NH_3_ + CO_2_	gas phase	solar energy	87 μmol g^−1^ h^−1^	Xia et al.^153^
Ti^3+^-TiO_2_/Fe-CNTs	N_2_ + CO_2_	Na_2_SO_4_, 0.5 M	300 W high-pressure Hg lamp	177.5 μmol L^−1^ g^−1^ h^−1^	Maimaiti et al.^152^
Mo(MnO_4_)_5_@Ce-BTC	NH_3_ + CO_2_	gas phase	visible light, 300 mW/cm^2^	70 μmol L^−1^ h^−1^	El-Shahat and Abdelhameed^137^
2D-CdS@3D-BiOBr	NO_3_^−^ + CO_2_	Na_2_SO_4_, 0.2 M	300 W xenon lamp,0.2 W cm^−2^	15 μmol L^−1^ g^−1^ h^−1^	Wang et al.^145^
Cu SA-TiO_2_	N_2_ + CO_2_	H_2_O	365 nm LED light, 433 mW cm^−2^	432.13 μg g^−1^	Li et al.^139^
Fe-TiO_2_/HZSM-5	NO_3_^−^ + CO_2_	KNO_3_, 1.6 Mm	high-pressure Hg lamp (250 W)	6.25 μg L^−1^ g^−1^	Srinivas et al.^151^
CdS-Bi_2_WO_6_	N_2_ + CO_2_	H_2_O	visible light (>365 nm)	10 μmol g^−1^ h^−1^	Wang et al.^161^

Generally speaking, in C–N coupling electrocatalytic systems, competing reduction processes (e.g., CO_2_ to CO, formate, or methane and N_2_ to NH_3_ or N_2_H_4_) hinder efficient C–N coupling and often lead to low urea selectivity. In such systems, chemical adsorption of the reactants is a key step to prompt the ultimate reaction, driven by effective interactions between the precursors and electrocatalytic active sites on the surface. Achieving the controlled, simultaneous reduction of the precursors within the same reaction environment is the key challenge of this approach. To overcome these obstacles and reach optimal urea production efficiency, an effective electrocatalyst should meet several significant criteria, including facilitating the strong adsorption and efficient activation of the reactants on its surface, enhancing the C–N coupling reaction between the desired intermediates and active sites, and lowering the energy barrier of the favorable reaction steps while suppressing competing side reactions. Furthermore, technical parameters, such as overpotential, Faradaic efficiency, current density, electrode stability, and C/N selectivity, should be precisely monitored and optimized to maximize the performance of urea electrosynthesis. The capability of directly controlling the reaction kinetics and selectivity in electrochemical systems is often difficult to achieve through traditional thermal or homogeneous catalytic methods. This enables the fine-tuning of the reaction pathways under moderate conditions, activating inert molecules and offering a better specificity in product generation, especially in complex multi-electron processes such as urea electrosynthesis. Also, these systems mostly exhibit reaction rates that favor the formation of higher-valued product distribution, which is a significant priority for scale-up and practical applications. This strategy eliminates the need for high-pressure and temperature circumstances to drive the reactions effectively while suppressing the emission of hazardous gases such as CO to the atmosphere. Hence, large-scale urea electrosynthesis could significantly contribute to reducing the effects of global warming by utilizing atmospheric CO_2_, thereby helping to mitigate climate change. It can also contribute to air purification in urban areas by converting toxic gaseous pollutants, such as CO and NO, frequently emitted by industrial activities and vehicles, into valuable chemical products. However, a significant challenge in electrocatalysis lies in designing and constructing highly stable electroactive surfaces that can act effectively as catalysts to assure long-term stability during the process. Another notable challenge in industrial electrocatalysis is the need for high-concentration electrolytes and high temperatures to ensure maximum yield, while the elevated temperatures compromise the stability of electroactive materials, specifically at the laboratory scale. This issue is further worsened by using high-concentration electrolytes, which provide a highly corrosive medium for conducting.

Regarding the photocatalytic approach, its eco-friendly nature, remarkable safety, and simplicity of operation make this strategy a more sustainable and adaptable alternative for urea production compared to the Bosch–Meiser process. In this context, the successful and efficient electron transfer to facilitate the multi-electron reduction of nitrogen and carbon source reactants toward C–N coupling occurrence is still crucial, but challenging. Substantially, the photogenerated electrons are the vital driving force to prompt the reduction steps in light-driven urea production systems. In a contrary manner, positively charged holes with strong oxidizing characters restrict the efficiency and selectivity of systems by adversely interfering with the simultaneous reduction processes. The catalyst construction plays a pivotal role here. For instance, with NO_3_^−^ precursor-contained systems, NO_3_^−^ rarely forms a single product; it produces a range of products, including NO_2_^−^, NH_4_^+^, and N_2_, with a selectivity strongly dependent on the catalyst type and reaction conditions. In the presence of H_2_O, photogenerated holes can oxidize H_2_O molecules and generate reactive ^·^OH radicals, which could promote some unfavorable side reactions such as cyclic reductant reformation of NO_3_^−^, which not only consume charge carriers that would otherwise accelerate reductive C–N coupling, but also re-oxidize reduced nitrogen intermediates back toward NO_3_^−^ or other less reduced states, decreasing the effectiveness of NO_3_^−^ utilization. As a result, urea selectivity in NO_3_^−^-based systems is fundamentally limited by competing oxidative pathways that divert electrons and holes toward useless byproducts, highlighting the need for rational catalyst design that suppresses these side occurrences while steering the selective C–N bond formation. Also, ^·^OH radicals could non-selectively oxidize some key organic intermediates, essential for urea synthesis. Another harmful consequence of ^·^OH activity is the dimerization of N_2_O to toxic N_2_O_4_, 100 ppm of which can cause pulmonary edema or even be fatal to humans. Therefore, photogenerated holes and ^·^OH species act as major sinks for reactive electrons, reducing selectivity and effective urea yield unless their formation and reactivity are carefully controlled through catalyst design or operating conditions. As another reactive species from photocatalysis, ^·^O_2_^−^ indicates a strong affinity for urea, as repeatedly supported by theoretical and experimental studies. These are among the undesired photooxidation and photoreduction pathways that are unavoidable in photocatalytic systems. These concerns can significantly influence scaling up photocatalytic urea synthesis systems for real-world applications. Additionally, employing robust and lasting illumination sources for large-scale utilization increases energy costs, making such systems less competitive with urea electrosynthesis. However, integrating both approaches offers a promising solution to overcome many of the technical challenges associated with each one toward developing practical systems for the large-scale production of high-quality urea.

## Photoelectrocatalytic synthesis of urea

The integration of photocatalytic and electrochemical strategies in PEC synthesis creates a powerful hybrid approach that utilizes the advantages of both methods. In contrast to photocatalysis, PEC systems rely on either an applied voltage or built-in electric fields to drive the separation of charge carriers.^29^^,^^179^ This reduces recombination-related energy losses and improves overall reaction efficiency. Compared to purely electrocatalytic systems, PEC configurations can directly utilize solar energy to lower the external electrical input required to drive thermodynamically demanding reactions such as C–N coupling. This dual capability creates a unique opportunity to modulate reaction pathways more precisely by adjusting both photonic and electrochemical parameters, including light intensity, applied voltage, and interfacial energetics.^8^ Although PEC technology is still in its early stages, this integrated approach offers clear advantages over using either method alone and shows strong potential as a more energy-efficient and flexible pathway for sustainable urea production. By adjusting parameters such as light intensity, applied voltage, and surface energetics, researchers can more precisely investigate reaction pathways. This level of control is particularly important for complex, multi-electron processes like producing urea from CO_2_ and nitrogen sources, where managing intermediates and selectivity is essential.

### Photoelectrocatalysis: from fundamental principles to urea synthesis applications

**Figure 8:**
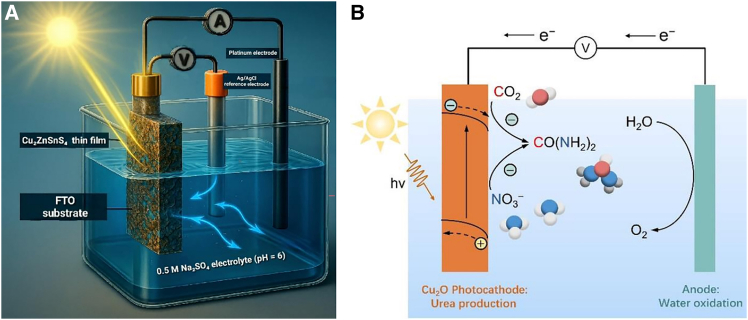
Urea synthesis routes (A) a simple photoelectrocatalytic system.^181^ Copyright 2024, espublisher. (B) Photoelectrosynthesis pathway of urea. Adapted with permission from ref.^29^ Copyright 2024, Wiley Online Library.

Photoelectrocatalysts combine the synergistic effects of photocatalysis and electrocatalysis by integrating the charge-transfer control of electrochemistry with the photoexcitation capabilities of photocatalysis.^180^ These catalytic systems, which utilize light and an applied electrical bias, can be constructed by immobilizing photoactive particles onto suitable conductive substrates that function as working electrodes. In addition to a working electrode, a reference electrode, and an auxiliary electrode are also essential components, enabling precise potential control and accurate monitoring of the electron transfer occurrences. Under the irradiation source, electron-hole charge carriers are generated on the surface of semiconductor-based electrodes. An applied electrical bias prevents the recombination of charge carriers; therefore, the separated charges can drive redox processes such as CO_2_ and NO_3_^−^ reduction (Figure 8A).^182^ The urea photoelectrosynthesis process (Figure 8B) focuses explicitly on accumulating photogenerated electrons and facilitating their kinetically and thermodynamically favorable transfer, aiming to reduce nitrogen and carbon sources simultaneously. Notably, a key advantage of the photoelectrocatalysis system is that the catalytic photoreduction and photooxidation half-reactions occur separately at the auxiliary and working electrodes. This spatial separation provides a versatile quantitative investigation of the desired reactions within an isolated medium.^183^^,^^184^ Photoelectrocatalysis, which combines the significant benefits of photocatalysis with the speed, simplicity, accuracy, and sensitivity inherent in electrocatalysis, is now a leading green technology for addressing environmental, energy, and chemical synthesis concerns.^185^

The efficiency of PEC systems depends on how effectively light is absorbed and how efficiently charge carriers (electrons and holes) are generated. The interaction of light with materials directly influences the generation of these carriers; therefore, photon management is crucial for overcoming thermodynamic limitations and improving the efficiency of activation reactions. Plasmonic materials, especially those with surface plasmon resonance (SPR), can generate energetic electrons and holes, so-called hot carriers. These hot carriers can then drive catalytic reactions, enhancing light-matter interactions and lowering activation barriers, thereby improving reaction rates and product selectivity. However, the efficient capture of these hot carriers, particularly holes, which have shorter mean-free paths than electrons, is the main challenge. Moreover, the effective separation and utilization of both hot electrons and holes is a key point to improving the performance of PEC systems. The catalyst design and its morphology also play a vital role in enhancing the PEC productivity. Materials such as Cu, Bi, and noble metals could be particularly prominent for activating CO_2_ and nitrogen precursors due to their unique optical properties and catalytic behavior at the nanoscale level. A precise optical and structural design is needed to maximize light absorption while minimizing shadow loss. Moreover, achieving strong light-matter coupling between plasmonic modes and dielectric resonances further enhances carrier generation and reaction dynamics, thereby improving selectivity and energy demand for processes such as urea synthesis.^186^

### Recent advancements in photoelectrocatalytic urea synthesis

Although urea conversion via photoelectrocatalysis has been frequently reported,^187^^,^^188^^,^^189^^,^^190^^,^^191^^,^^192^ the literature specifically addressing PEC urea synthesis remains limited. Zheng et al.^193^ developed a series of bi-metallic Cu-Ti dual active sites on TiO_2_ to enhance the photoelectrochemical C–N coupling reaction between CO_2_ and NO_3_^−^, aiming at urea synthesis. Compared to pure TiO_2_, the Cu-Ti/TiO_2_ catalyst showed a 2.66-fold enhancement in urea production yield (52.45 μmol L^−1^ h^−1^) with a Faradic efficiency of 68% under ambient conditions and an applied voltage of −0.5 V. The free energy formation of various intermediates on the materials was theoretically calculated using DFT, with experimental insights obtained from *in situ* DRIFTS measurements. The high performance arises from proximity-enabled C–N coupling, in which Cu sites strongly adsorb and reduce CO_2_ to ∗CO while adjacent Ti sites selectively generate ∗NH_2_ from NO_3_^−^, allowing direct ∗CO–∗NH_2_ coupling with a lower energy barrier than competing reactions. The PEC system provides a unique advantage by using photogenerated holes to dynamically suppress side reactions such as CO desorption and NH_3_ formation, thereby steering selectivity toward urea. This preferential binding suppressed competing reactions while enabling selective C–N coupling for urea formation. Although this work presents an insightful and convincing strategy for PEC urea synthesis, both reproducibility and scalability remain significant challenges. Reproducing the results requires precise control over single-atom Cu placement and Cu–Ti spacing, which are highly sensitive to synthesis conditions and difficult to achieve without advanced characterization tools. Scaling up further demands the fabrication of large, stable photoelectrodes that preserve single-atom integrity, provide uniform light exposure, and operate reliably over long periods. Additionally, the complexity and cost of PEC reactors, uncertainties around long-term durability, reliance on NO_3_^−^ feedstocks, and the difficulty of separating urea at low concentrations represent significant barriers to practical implementation.

**Figure 9:**
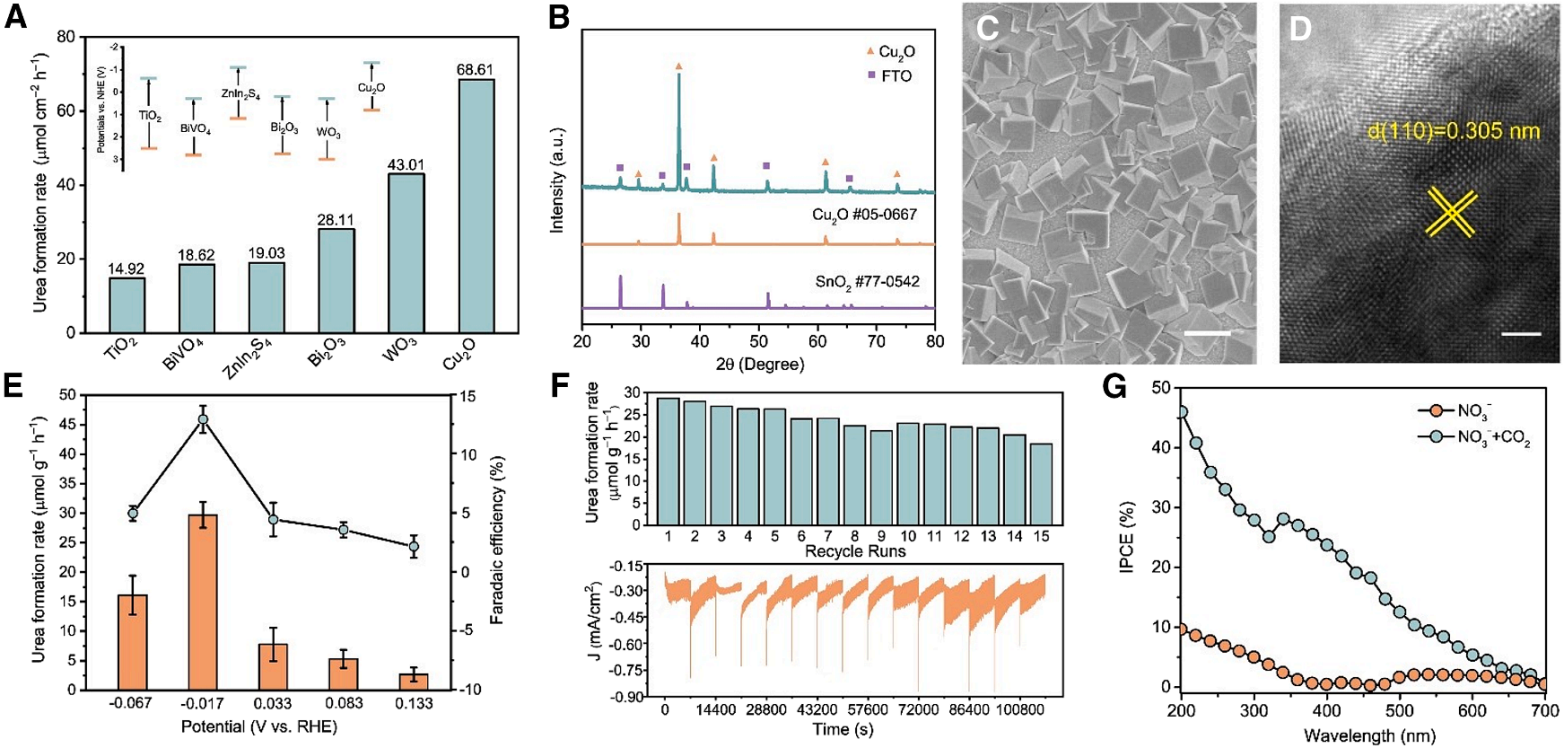
Photoelectrocatalytic activity and physicochemical properties of cubic Cu_2_O nanoparticles for urea synthesis (A) Urea production yield with various semiconducting materials as the photoelectrode (band positions are pointed out in the inset). Reaction medium: KNO_3_ solution (0.1 M) with a constant CO_2_ flow rate of 20 mL/min and AM 1.5 G illumination source. (B) XRD results. (C) SEM images with scale bars of 5 μm. (D) TEM images with scale bars of 2 nm. (E) Urea production yield besides the FE values with changing the external potentials on Cu_2_O. (F) Recyclability experiments, and (G) IPCE curves for Cu_2_O in the presence and absence of carbon dioxide. Adapted with permission from ref.^29^ Copyright 2024, Wiley Online Library.

In another work, Li et al.^29^ developed an efficient Cu_2_O-based photocathode with cubic morphology for the photoelectrochemical urea synthesis from NO_3_^−^ and CO_2_. The urea production yield on this photoelectrode reached 29.71 μmol g^−1^ h^−1^ with a Faradaic efficiency of 12.9% at a minimal external bias of −0.017 V vs. RHE. A comparative investigation of the catalytic activity of pure semiconductors highlighted the strong potential of Cu_2_O for urea formation under photoelectrosynthesis conditions (Figure 9A). The phase purity and well-defined cubic morphology of the Cu_2_O photocatalyst were also confirmed by XRD analysis (Figure 9B) and microscopy images (Figures 9C and 9D), demonstrating the successful synthesis of pure phase without CuO impurities and precise morphological control of Cu_2_O. Figure 9E reveals a strong potential-dependent behavior, where applied bias modulated urea production rates across an order of magnitude (5–29 μmol g^−1^ h^−1^) while simultaneously affecting Faradaic efficiency. The observed voltage sensitivity indicates that competing reduction pathways become dominant at higher overpotentials, suggesting an optimal potential range for selective urea formation. Therefore, precise control of the applied potential is critical to enhance both urea yield and electron utilization. This fact shows the importance of optimizing the rational correlation between the illumination source intensity, external potential, and resulting urea formation rate. The observed decline in catalytic activity during the initial recycling runs (Figure 9F) was primarily caused by the photochemical reduction of Cu_2_O to metallic copper (Cu^0^) under operational conditions, emphasizing the need for further improved strategies to stabilize the photoelectrode surface composites. Figure 9G presents the incident photon-to-current efficiency (IPCE) results, which illustrate how effectively photon energy contributes to electric current generation. As shown in the plots, the higher IPCE values correspond to improved PEC performance. This effect was particularly pronounced in the 350–500 nm wavelength range, highlighting the excellent light-harvesting capability of Cu_2_O and its efficient photogenerated charge migration under illumination. The key innovation of this research is the use of solar energy to generate an internal photovoltage, thereby significantly reducing electrical energy input. In fact, exposure to light boosts urea production by nearly 9 times compared to conventional dark electrocatalysis. In addition, the experimental methodology in this study is reproducible primarily because it employs commercially available Cu_2_O and rigorously validated analytical techniques. However, achieving consistent results faces challenges, including photocorrosion, partial reduction of Cu_2_O to metallic Cu during operation, and strong sensitivity to light-source characteristics and PEC cell configuration. Scaling up the process presents even greater difficulties, including inherently low production rates that would require very large photoreactor areas, the need to develop stable and conductive protective coatings to prevent the degradation of the Cu_2_O photocathode, complex reactor designs to ensure uniform illumination and efficient mass transport, and the necessity of balancing overall energy efficiency with capital and operational costs.

TiO_2_-based PEC materials incorporating Ru, Cu, and Zn have been recently synthesized by a combined hydrothermal-photodeposition approach and successfully employed for the synthesis of urea from CO_2_ and NO_2_, under illumination by a 300 W xenon lamp.^194^ The results indicated that the optimal 5% of Ru in Ru-TiO_2_ photocatalyst led to 50.58 μmol g^−1^ h^−1^ with the Faradaic efficiency of 16.66% (1.65 times higher than the Faradaic efficiency over pure TiO_2_) at an external bias of −0.1 V vs. RHE. In addition to improving the light-harvesting capabilities of pure TiO_2_, Ru atoms could also facilitate the efficient transfer of photogenerated electrons, thereby promoting C–N coupling and urea formation.

Despite the limited number of publications currently available on the topic, the simultaneous utilization of the major global warming contributors like CO_2_ and nitrogen-containing pollutants such as NO_3_^−^ and NO_2_^−^ remains a substantial contaminant in the drinking H_2_O resources worldwide, alongside the unique versatility aspects of the photoelectrochemical systems, which increase the potential for increased research activity in this field in the near future.

### Analytical strategies for urea yield assessment in photoelectrocatalytic systems

**Figure 10:**
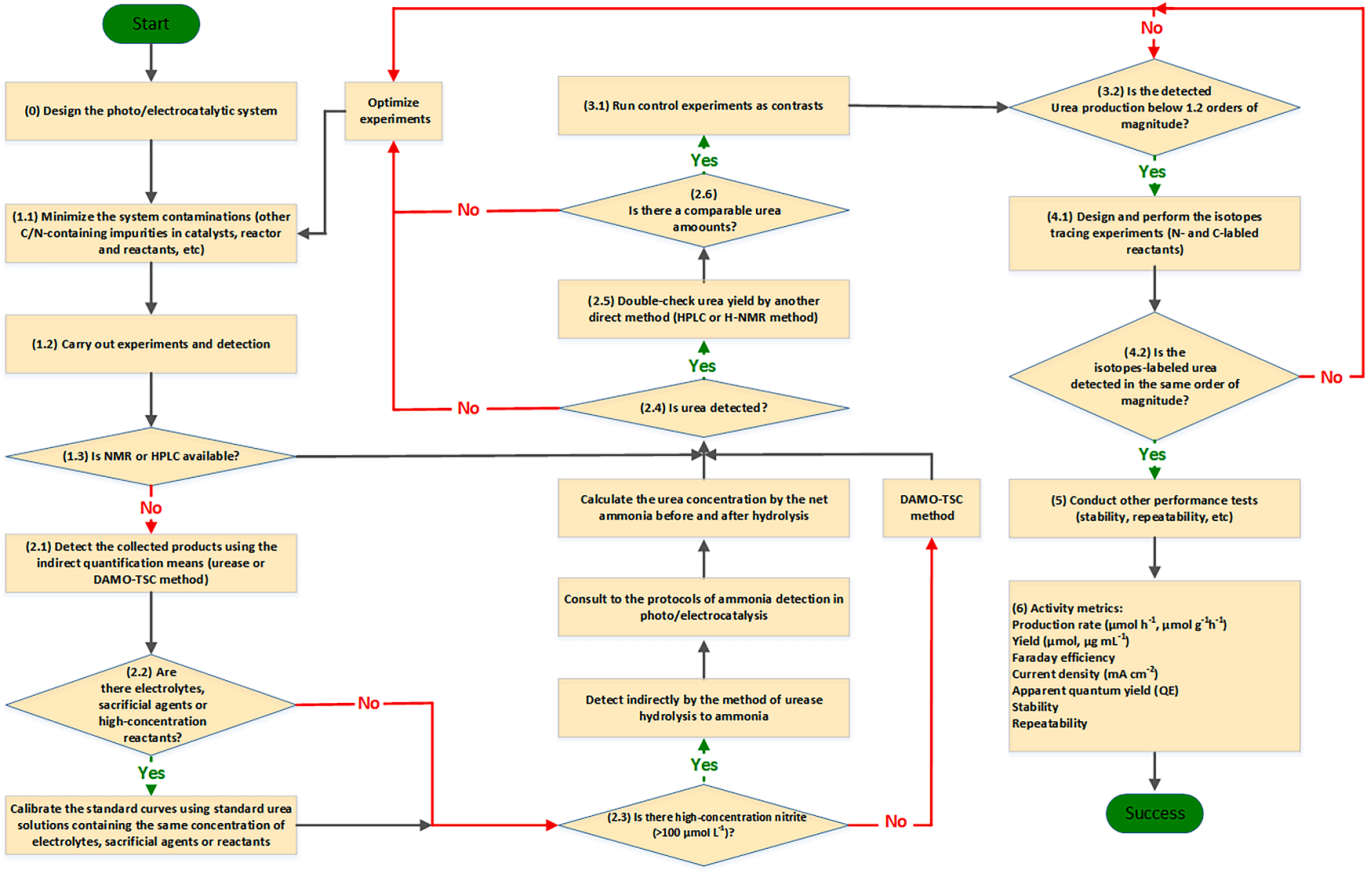
The suggested protocol for urea determination from photoelectrocatalytic synthesis routes Adapted with permission from ref.^195^ Copyright 2025, Wiley Online Library.

Li et al. were the first to propose a systematic protocol for quantifying urea and other products generated from the PEC synthesis under ambient conditions.^195^ Their work systematically evaluated urea detection approaches and ultimately developed an accurate analytical method specifically tailored for PEC systems (Figure 10). A key requirement highlighted in this protocol is the strict elimination of background contamination from carbon- and nitrogen-containing species, which is essential to avoid false-positive urea signals. Beyond contamination control, the authors emphasized the necessity of carefully designed control experiments, including catalyst-free, reactant-free, dark, light-only, and bias-only conditions, to unequivocally confirm the true origin of the detected urea. If the target product (urea) and/or a similarly effective alternative cannot be produced, the PEC system loses its practical value for urea synthesis. Furthermore, isotopic labeling experiments with ^13^CO_2_ and/or ^15^NO_3_^−^ were recognized as essential techniques for confirming C–N coupling pathways and excluding contributions from unintended sources or solution-phase reactions. Ultimately, the use of standardized performance benchmarks, normalized to catalyst mass or area, reaction time, and photon input, is essential for enabling meaningful comparisons among different PEC systems and for accurately evaluating their practical viability. It should be noted that discrepancies among urea quantification methods can substantially affect the comparability of reported yields across different studies. In particular, colorimetric UV–Vis-based assays, although simple and widely adopted, are susceptible to chemical interferences from nitrogen-containing intermediates, electrolyte components, and side products, which may lead to systematic overestimation of urea yields. As a result, urea production values derived solely from colorimetric methods are not always directly comparable to those obtained using more selective analytical techniques. In contrast, LC-MS provides higher analytical specificity by separating urea from coexisting species before detection, thereby minimizing false-positive signals and improving yield reliability, albeit at the expense of higher cost and experimental complexity. NMR spectroscopy, especially when combined with isotopic labeling (^13^CO_2_ and/or ^15^NO_3_^−^), serves as a powerful confirmatory tool that enables unambiguous identification of urea and validation of C–N coupling pathways, though its quantitative sensitivity is generally lower than that of LC-MS. Therefore, meaningful benchmarking of PEC urea synthesis requires transparent reporting of analytical methodologies, calibration procedures, and detection limits, as well as complementary validation using at least two independent techniques whenever possible.

The PEC performance of a system is commonly evaluated using Faradaic efficiency and urea production yield. However, these metrics alone are insufficient without accurate and reproducible analytical quantification. Therefore, precise, highly analytical techniques with minimal bias and maximum reproducibility are required. In PEC systems, urea yield alone is insufficient to evaluate performance and must be assessed by metrics such as product selectivity, carbon and nitrogen mass balance, catalyst stability, and time-dependent yield behavior. Additionally, photon-related parameters, including light intensity, wavelength dependence, and photon-to-urea efficiency, are necessary to confirm actual photo-driven activity.

Liquid chromatography-mass spectroscopy (LC-MS) is a reliable, though costly, technique for urea detection in photoelectrosynthesis methods. Due to the distinct mass differences between urea and byproducts generated from photoelectrochemical or electrochemical side reactions, the LC-MS technique effectively isolates and quantifies urea while minimizing interferences from the byproducts based on this criterion.^196^ As with other chromatography techniques, selecting suitable mobile phases, flow rates, and calibration standards is critical for achieving accurate analysis. In LC-MS, the individual components are first separated and then ionized for mass spectrometric detection. The separated ions are subsequently sorted by their mass-to-charge (m/z) ratio. The signal intensity at each m/z value corresponds to the relative abundance of specific species in the product mixture. Several researchers have developed reliable analytical methodologies utilizing this robust technique for the simultaneous determination of urea and other C–N-containing compounds in the complex reaction mixture.^197^^,^^198^

Urea can be analyzed simply by UV-Vis spectroscopy after pretreatment with either of two common chromogenic reagents, urease-indophenol blue or diacetyl-monoxime (DAMO).^199^^,^^200^ Urea (in the earlier method) and its hydrolysis product, NH_3_ (in the subsequent approach), are each used directly to form colored complex solutions, which are then analyzed by UV-Vis spectrophotometry to determine their concentrations. The resulting data can be easily correlated with the urea yield in the reaction mixture. Both protocols offer a straightforward, user-friendly approach for the rapid detection of urea in photoelectrochemical reaction media; however, chemical interferences during the complexation steps remain unavoidable.^195^

To ensure the accuracy of urea yield data obtained by UV-Vis spectroscopy, NMR spectroscopy can be used as a robust analytical technique. In this technique, DMSO-*d*_6_ and D_2_O are the most commonly used reagents, enabling rapid urea detection by ^1^H-NMR, which benefits from hydrogen-deuterium exchange.^201^ Since photoelectrosynthesis systems contain carbon-based compounds, ^13^C NMR is an effective characterization tool, offering strong capabilities for analyzing reaction mixtures. Herein, researchers employ ^13^CO_2_ as the primary carbon source to produce ^13^C-urea through PEC synthesis.^195^ Table 4 compares urea quantification methods, outlining their key advantages and disadvantages.

**Table 4:** Advantages and disadvantages of urea quantification methods^79^

Analytical method	Sensitivity and selectivity	Key advantages	Main limitations	Recommended role
HPLC/HPLC-MS	very high	excellent separation of urea from byproducts; suitable for complex media; enables multi-product analysis	high cost; limited accessibility; requires careful calibration	primary quantitative method; selectivity and mechanistic studies
UV–Vis (Indophenol/DAMO)	moderate	simple, rapid, low cost; suitable for routine screening, few influencing by-products	susceptible to chemical interferences and false positives; possible overestimation	preliminary quantification; must be validated by other techniques.
^1^H NMR	high	direct urea identification; minimal interference; good reproducibility, isotope labeling	lower sensitivity than LC-MS; requires deuterated solvents, and has quantification difficulties	quantitative validation and cross-checking
^13^C NMR (with^13^CO_2_)	high (mechanistic)	direct evidence of carbon incorporation confirms proper C–N coupling	expensive isotopes; longer acquisition time	mechanistic confirmation and isotope tracing

### Mechanistic insights into photoelectrocatalytic urea synthesis via *in situ* FTIR analysis

**Figure 11:**
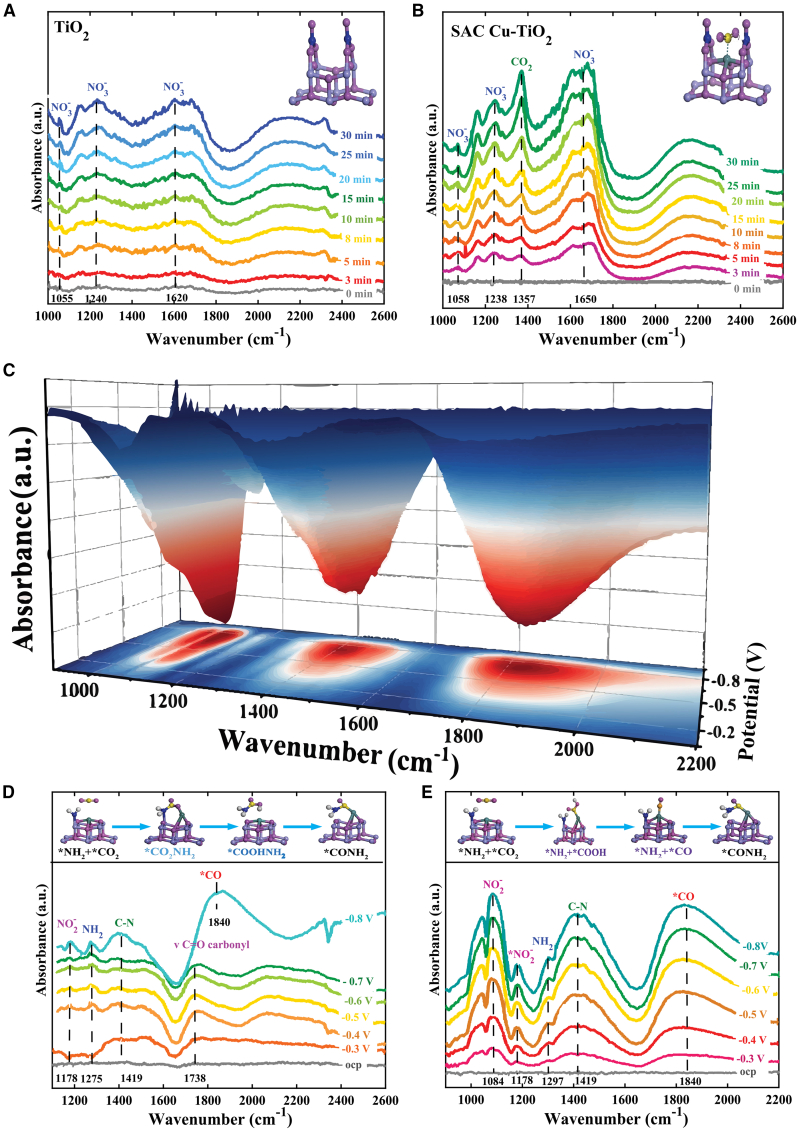
FT-IR detection of adsorbed CO_2_ and nitrate on different structures FTIR analysis of the adsorption of nitrate and CO_2_ on TiO_2_ (A) and single-atom doped SAC Cu-TiO_2_ (B). The results of 3D *in situ* FTIR for urea photoelectrosynthesis on the doped photocatalyst (C). *In situ* FTIR investigation of the reaction intermediates through urea electrosynthesis (D) and photoelectrosynthesis (E). Adapted with permission from ref.^193^ Copyright 2024, Elsevier.

The three main sequential stages of a PEC reaction include photon absorption by the photoactive component, leading to electron-hole generation within and/or on the surface, charge carriers migration through the photo/electro-active regions, and adsorption and reaction of reactants and byproducts on the photoelectrode surface to form the selective compounds on the surface of the photoelectrode, which are then desorbed into the medium for instrumental analysis.^202^ Research on photocatalysis has focused extensively on understanding charge-transport mechanisms and on enhancing photocatalytic efficiency through morphological and structural modifications.^203^ To effectively elucidate product selectivity and reaction pathways, *in situ* analytical techniques, particularly FTIR spectroscopy, are crucial. This technique provides molecular-level insights into surface reactions, intermediate formation, and degradation pathways during photocatalytic processes.^204^ Zheng et al. used *in situ* FTIR to reveal that the NO_3_^−^ species (characterized by bands at 1055,1240, and 1620 cm^−1^) initially adsorb on TiO_2_ active sites while CO_2_-related species (1640 cm^−1^) are anchored to Cu single-atom sites in a TiO_2_/Cu-SAC system during NO reduction (Figures 11A and 11B).^193^ The FTIR peaks at 1275, 1419, and 1738 cm^−1^ are assigned to the ∗NH_2_ intermediate, C–N bonding formation, and carbonamide intermediate (∗H_2_NCOOH), respectively, implying coupling between ∗NH_2_ and CO_2_ forms ∗H_2_NCOOH intermediate, which subsequently undergoes multi-step conversion to urea. Despite spectroscopic evidence suggesting a ∗NH_2_/CO_2_ coupling pathway indicated by peaks at 1275 cm^−1^ [∗NH_2_], 1419 cm^−1^ [C–N], and 1738 cm^−1^ [∗H_2_NCOOH]), the authors reported the detection of NH_3_ and adsorbed carbon monoxide (∗CO) peaks in their results (Figures 11C–11E), which highlights unresolved selectivity challenges in photocatalytic urea synthesis. Therefore, even though Cu/Ti sites significantly lower the barrier for urea formation, these side reactions still consume a portion of the critical ∗CO and ∗NH_2_ intermediates. Suppressing these pathways is key to further increasing urea Faradaic Efficiency (FE). A targeted defect-engineering strategy can be employed to suppress CO and NH_3_ side reactions and promote urea formation by simultaneously tuning the adsorption and reactivity of key intermediates on Cu–TiO_2_ catalysts. By introducing oxygen vacancies into the TiO_2_ support near isolated Cu atoms, the electronic environment of the Cu site can be adjusted; therefore, the key ∗CO intermediate binds more strongly and is less likely to desorb as CO gas. These vacancies donate electron density to the Cu, improving back-donation into the antibonding orbitals of CO and keeping ∗CO on the surface long enough to participate in C–N coupling, without site poisoning, thereby retaining ∗CO for C–N coupling. At the same time, oxygen-vacancy-related Ti^3+^ sites on TiO_2_ can be used to inhibit hydrogenation of ∗NH_2_ on Ti sites to NH_3_, thereby stabilizing ∗NH_2_ in a configuration less favorable for further hydrogenation by increasing the energy barrier to proton attack and extending its surface lifetime. A more integrated strategy is to create a defect-rich nano-environment in which oxygen vacancies are arranged to bring ∗CO on Cu and ∗NH_2_ on neighboring Ti sites into closer electronic and geometric contact, facilitating electron transfer, improving orbital overlap, and stabilizing the transition state for C–N coupling. Collectively, these defect-induced effects are expected to lower the activation barrier of the rate-determining C–N coupling step (CONH_2_ + NH_2_ → urea), kinetically favoring urea formation over desorption or hydrogenation pathways.

Hence, the PEC pathway for urea generation in this system was proposed as follows, which was in good agreement with DFT calculations.

(Equation 40) CO2∗+NH2∗→NH2∗+COOH∗→NH2∗+CO∗→CONH2∗

In another study, *in situ* EPR was employed to identify the reaction mechanism of urea photoelectrosynthesis over a Cu_2_O photocathode, considering the potential involvement of radicals.^29^ No radicals were detected during the reaction, suggesting that the process may not have obeyed a radical-mediated pathway. However, when persulfate was introduced as an electron scavenger, the urea formation rate was significantly suppressed, indicating that photogenerated electrons play a crucial role in driving the reaction. Moreover, photo-assisted online DEMS results revealed that NO_2_ serves as the key intermediate derived from the nitrogenous source. In addition, *in situ* FTIR analysis detected the characteristic peaks corresponding to CO (2073 cm^−1^), C–N bonding (1415 and 1452 cm^−1^), and –NH_2_ (3440 cm^−1^), providing strong evidence for the proposed urea formation mechanism outlined in the following equations:

(Equation 41) NO3−+2H++e−→NO2∗+H2O

(Equation 42) NO3−+hv→NO2∗+O·−

(Equation 43) CO2+2H++2e−→CO∗+H2O

(Equation 44) CO∗+NO2∗→CONO2∗

**Figure 12:**
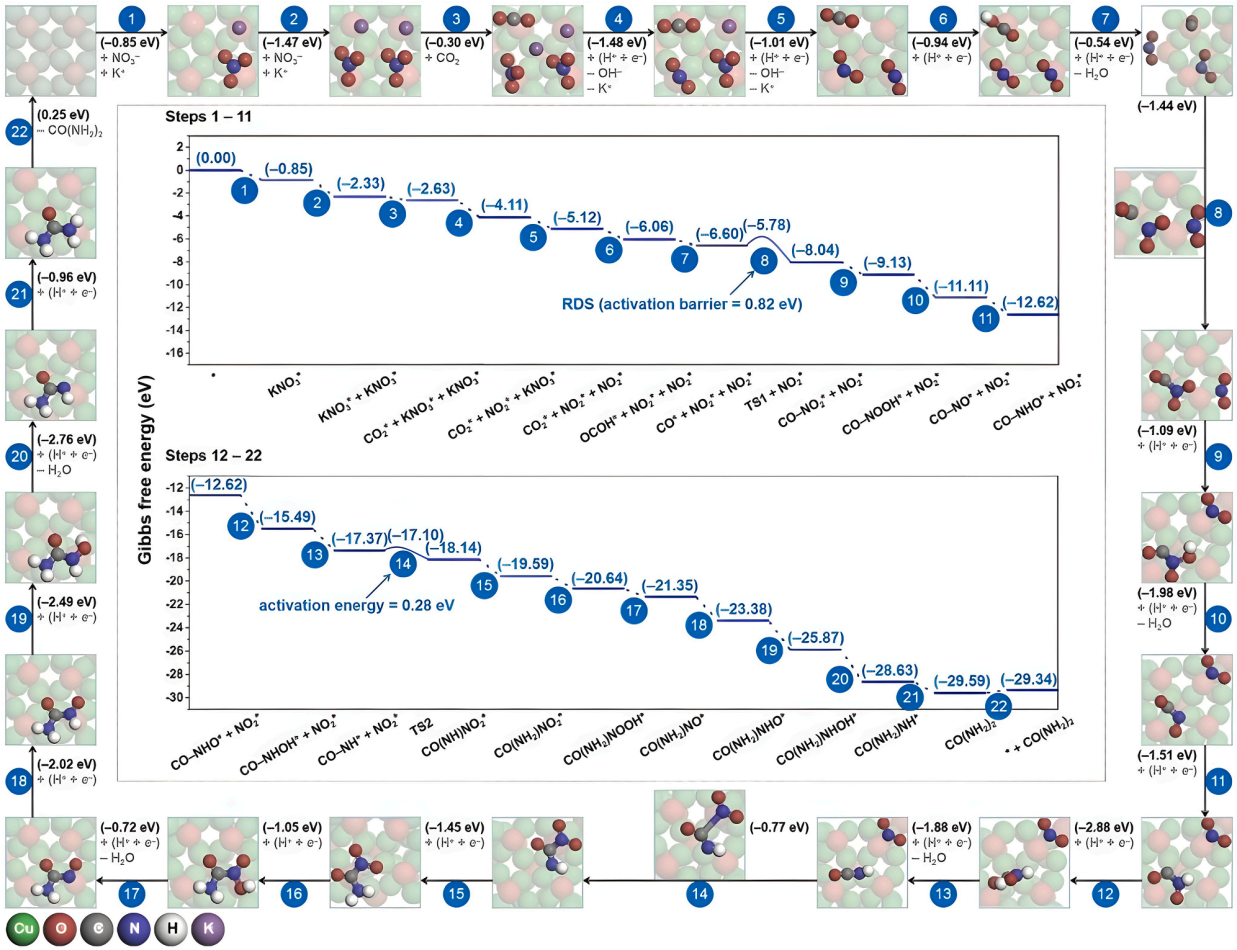
Theoretical evidence on the mechanism of urea formation through simultaneous reduction of nitrate and carbon dioxide over a Cu_2_O photoelectrode The external cycle illustrates the suggested pathway. The elementary steps of the process are marked with blue values. The optimized geometries are exhibited with the color code indicated below, and the inset is the analogous Gibbs-free energy diagrams alongside the activation energy barriers. Adapted with permission from ref.^29^ Copyright 2025, Elsevier.

The final intermediate, ∗CONO_2_, undergoes sequential reduction (hydrogenation) to form urea, as supported by the DFT calculations and Gibbs free energy diagrams (Figure 12). Their findings revealed that the first coupling between ∗CO and ∗NO_2,_ followed by the second C–N coupling between the ∗CO–NH and ∗NO_2,_ were recognized as the rate-determining steps in the reaction mechanism.

## Economic prospects and industrial challenges in urea photoelectrosynthesis

PEC urea synthesis integrates the photocatalytic and electrochemical processes to convert carbon and nitrogen sources, thereby improving charge separation and reaction efficiency under an applied potential. PEC systems offer environmental benefits such as preventing secondary pollution, lowering operational costs, and reducing treatment duration, which are significant advantages in various photoelectrocatalysis applications.^205^ Although PEC provides a promising route for sustainable urea production, its industrial feasibility depends on overcoming significant economic and engineering challenges.^206^ Unlike the Haber–Bosch process, which benefits from over a century of large-scale deployment, PEC systems are currently limited by technical and operational obstacles. To assess the economic feasibility of PEC urea synthesis, a Techno-Economic Analysis (TEA) is necessary, focusing on key factors, including capital and operational cost, efficiency, energy consumption, and scalability.^207^ Considering capital costs, PEC systems face substantial expenses due to the use of expensive noble metal electrode materials and complex reactor designs, while the Haber-Bosch process benefits from economies of scale and optimized infrastructure, which lower its capital costs. In terms of operational costs, PEC systems face varying costs depending on renewable energy availability, long-term stability and replacement of the catalyst, and system maintenance, while the Haber-Bosch process is energy-intensive and relies heavily on natural gas but benefits from stable and highly optimized infrastructure.^208^

Regarding efficiency and energy consumption, PEC systems utilize solar energy to drive reactions at ambient temperatures and pressures, reducing external energy needs; however, current PEC systems are less efficient than the Haber-Bosch process. Although PEC consumes less energy, inefficiencies in catalyst design and light absorption limit its performance.^209^

Considering scalability, PEC systems suffer from significant scalability challenges, such as mass-transfer limitations, uniform light distribution, and maintaining homogeneous potential fields across large electrode areas. In addition, designing a mechanically robust, weather-resistant reactor, challenges of scaling up photoelectrode production, and ensuring system durability are also costly. In contrast, the Haber-Bosch process is highly optimized for large-scale NH_3_ production, benefiting from industrial-scale infrastructure.

To further support the comparison, data from research and pilot-scale projects on H_2_ and NH_3_ production were examined alongside those from the Haber-Bosch process. A relevant case study is the European Commission-funded project on solar-driven NH_3_ production, led by companies Repsol and Enagás.^210^ The project aims to produce 100 kg of clean hydrogen per day using solar energy, with plans to scale up to 200 tonnes per year by connecting to the electricity grid. As another case study, Tsang’s research team developed an innovative renewable power-to- NH_3_ pilot project in collaboration with Siemens, designed to produce 11 tonnes of green NH_3_ annually using a 13 kW wind-powered proton-exchange membrane (PEM) H_2_O electrolyzer.^211^ This project highlights the feasibility of integrating renewable energy sources with electrochemical processes for sustainable chemical production. It demonstrates the challenges of scaling such systems, particularly in terms of efficiency and cost, while emphasizing the importance of designing systems that are robust, scalable, and capable of continuous operation.

These case studies reveal both the potential and challenges of PEC systems, particularly in terms of cost and efficiency. Although PEC systems offer environmental benefits, including the potential for carbon-neutral urea production when powered by renewable energy, its high initial costs and limited scalability prevent them from competing with the Haber-Bosch process at present. However, with advancements in catalyst development and reactor design, PEC could become a competitive method in the future, especially if material costs are reduced and efficiency is improved.^212^

To compare PEC and Haber-Bosch systems, specific benchmarks for efficiency, cost, and scalability are necessary. PEC systems must achieve 10–20% solar-to-urea efficiency, compete with Haber-Bosch, which operates at 60–70% energy efficiency on a lower heating value basis, reaching up to ∼80% under optimized conditions,^213^ though it relies on natural gas and has significant environmental drawbacks.^214^

The current PEC systems typically exhibit very low efficiencies (≪1%),^215^^,^^216^ and improvements are expected with advancements in catalysts and reactor designs. A key challenge of PEC systems is electrode lifetime; stability targets reported for solar fuel and electrolysis systems span from >10^3^ h in laboratory demonstrations to >10^4^–10^5^ h for practical devices, corresponding to approximately year-scale operation (∼1–2 years) for early commercial viability,^217^^,^^218^ whereas the Haber-Bosch process has highly stable reactors designed for long-term continuous operation. Reducing material costs by a factor of 5–10 through eliminating noble metals and utilizing more earth-abundant materials, such as Cu, Fe, or Ni catalysts, would be a critical goal. In comparison, Haber-Bosch benefits from lower material costs due to its reliance on natural gas and inexpensive iron-based catalysts. PEC urea production must reduce costs to below $500 per ton and approach current market prices for industrial urea (∼$300–500 t^−1^) to remain competitive with the Haber–Bosch process, whose costs range from $200 to $500 per ton depending on natural gas prices.^214^^,^^219^^,^^220^

However, PEC systems offer significant environmental benefits, such as the potential for carbon-neutral urea production when powered by renewable energy like solar, making it a cleaner alternative to fossil fuel-based methods. In contrast, the Haber-Bosch process has a high environmental impact due to its reliance on fossil fuels, leading to substantial CO_2_ emissions and energy consumption. Despite its environmental impact, the Haber-Bosch process remains dominant due to its cost, efficiency, and established infrastructure. However, advancements in PEC catalysts and reactor design could make it a competitive method in the future, especially if it can reduce material costs and improve efficiency.^221^

For the PEC urea production to become economically feasible, research must go beyond just improving catalytic efficiency and tackle the practical challenges associated with scaling the technology. This requires a focused effort on developing durable, earth-abundant catalysts, creating innovative reactor designs to enhance mass transport and light utilization, and conducting Techno-Economic Analysis (TEA) using data from continuous, long-term prototype operations instead of short laboratory tests.^222^

Integrating PEC technologies with circular economy principles can significantly enhance sustainability by utilizing wastewater containing NO_3_^−^ as a nitrogen source for urea synthesis. This approach not only helps address NO_3_^−^ pollution but also supports agricultural needs while minimizing environmental impact. When powered by renewable energy, such as sunlight, the process becomes even more sustainable, reducing carbon emissions.^223^ This approach enhances the overall feasibility and impact of PEC technologies in real-world applications while contributing to the global shift toward more sustainable, circular industrial practices. Efficient urea production from NO_3_^−^ containing wastewater requires coordinated optimization of reactant selection, catalyst design, reaction conditions, and practical implementation. A key factor in this approach is the design of electrocatalysts, which must selectively facilitate C–N coupling while preventing undesirable reactions. Achieving selectivity in this process often requires dual- or multifunctional active sites that can activate both CO_2_ and NO_3_^−^. This can be accomplished through atomic-level strategies, including single- and dual-atom catalysts (DACs), defect engineering to modify the electronic structure and stabilize intermediates, and the use of bimetallic or compound catalysts. Additionally, structural control techniques such as facet engineering, heterostructures, and porous frameworks are essential.^224^

Beyond the catalyst itself, the characteristics of wastewater, including pH, NO_3_^−^ and bicarbonate levels, and alkali metal cations, significantly affect reaction kinetics and selectivity. Finally, ensuring stable, long-term operation and robust performance in real wastewater environments rather than idealized electrolyte conditions remains a major challenge.^225^

## Perspectives on the photoelectrocatalytic urea synthesis: future research direction

The development of catalysts that are highly selective, active, nontoxic, and cost-effective is crucial for PEC urea synthesis.^52^ The integrated design emphasizing novel structures, molecular modeling, advanced characterization techniques, and novel synthesis methods will help regulate the selectivity and efficiency of the catalysts. As previously noted, a typical PEC system primarily consists of a photoelectrode comprising a semiconductor and cocatalysts. The photoelectrode converts solar energy into charge carriers, generating photovoltage to drive redox reactions, with electrons and holes directing reduction and oxidation processes, respectively.^226^ Recent advances in photoelectrode design have focused on enhancing light absorption, promoting efficient charge separation, and optimizing cocatalyst integration. Designing catalysts for PEC urea synthesis should facilitate cooperative activation mechanisms, promote the reactions to target specific reaction pathways, maintain high selectivity by suppressing side reactions such as H_2_ evolution and the complete conversion of precursors to urea, overcome mass-transfer limitations that hinder the efficient delivery of gaseous reactants like CO_2_ and N_2_ to the solid–liquid–gas reaction interface, and stabilize crucial intermediates. Employing advanced light absorbers offers a straightforward and impactful approach to improving solar-to-chemical conversion in PEC systems. However, effectively integrating light absorbers, cocatalysts, and counter electrodes remains a major engineering challenge. Significant efforts are required in photoelectrode design to optimize light harvesting and charge utilization for target reactions, as well as in overall system engineering to ensure that power output approaches the maximum achievable under real operating conditions. Understanding reaction mechanisms, especially in systems that produce more complex products, is another challenge.^227^ This mechanistic understanding is the vital bridge that connects the design of the catalyst to its ultimate performance. Although urea selectivity in many PEC systems remains below 50%, combining advanced real-time characterization techniques with thorough kinetic studies and control experiments provides a diagnostic framework for tracing reaction pathways and identifying the fundamental steps that determine selectivity and efficiency. Fortunately, various *in situ* and semi-in situ characterization methods have been applied in PEC research, offering valuable mechanistic insights.^228^ Moreover, the long-term stability of PEC systems under continuous light exposure and applied voltage remains limited, and substantial work is required to scale these technologies and demonstrate their performance under real-world operating conditions.^229^

In addition to catalyst issues, the most significant engineering barriers to scaling up PEC urea synthesis lie in moving from controlled laboratory setups to industrial flow-reactor systems. Key challenges include developing gas-diffusion electrodes^230^ that can efficiently deliver CO_2_ to the reaction sites and creating durable membrane/electrode assemblies that can withstand long-term photoelectrochemical operation.^231^ These obstacles remain crucial to making the technology practical on an industrial scale. Most PEC urea studies are conducted in batch reactors or small-scale cells, where factors such as reactant diffusion, light exposure, and catalyst/electrolyte contact can be easily controlled. However, scaling up to industrial continuous flow reactors introduces several complex challenges. Efficient mass transport, requiring constant delivery of CO_2_ and nitrogen precursors and effective removal of products to avoid local concentration gradients, becomes critical. Achieving uniform light distribution across large electrode surfaces is also difficult, as uneven irradiation can reduce charge-carrier generation, lowering urea production efficiency.

Additionally, industrial reactors must manage heat accumulation resulting from light absorption to ensure stable operation and prevent catalyst degradation. Finally, optimizing flow dynamics such as turbulence, pressure drops, and residence time is essential to maintain reaction kinetics while minimizing side reactions.^232^ Gas-diffusion electrodes (GDEs) are crucial to PEC urea synthesis because they help deliver CO_2_ directly to the catalyst, overcoming its low solubility in H_2_O and boosting reaction rates. However, designing effective GDEs is challenging because they require a balance of hydrophobic and hydrophilic properties to allow gas flow without flooding, maintain good ionic contact, and remain structurally stable during long-term operation. Scaling up adds another layer of difficulty, as producing large, uniform, and mechanically durable electrodes is technically demanding.^233^ Durable membrane/electrode assemblies are key components in PEC reactors, combining catalysts, electrodes, and ion-conducting membranes to enable efficient charge transfer. Designing a robust membrane electrode involves several engineering challenges, including mechanical stability under repeated thermal cycles, light irradiation, and electrolyte flow; chemical durability against oxidative degradation; and resistance to fouling by byproducts. Additionally, the membranes must balance ion conductivity with selectivity to maintain efficient ion transport. Integrating the membrane/electrode with gas-diffusion electrodes further increases complexity, requiring both components to be maintained in close contact and to maintain structural integrity during operation.^234^ At the level of the industrial system, energy efficiency requires carefully balancing light input, applied voltage, and chemical output to maintain economic feasibility.^235^ Industrial systems may also need integration with CO_2_ capture, gas compression, and urea separation units to operate effectively.^229^ Furthermore, long-term reliability requires materials and designs that resist fouling, degradation, and catalyst loss, thereby reducing maintenance and operational downtime.^236^

### Machine learning for electrocatalytic urea synthesis

Machine learning (ML) has become a powerful tool for screening catalysts in PEC urea synthesis. This is driven by advances in algorithms and their application, addressing the inherent complexity of PEC reactions, the wide variety of potential catalyst compositions and structures, and the urgent need to develop efficient, scalable systems quickly.^237^ The data collection, descriptor generation and selection, algorithm selection, and result evaluation are the key steps in constructing an ML model.^238^

Machine learning contributes to the development of photoelectrochemical catalysts in two key ways. First, it enables the construction of robust low-dimensional descriptors that effectively describe catalytic activity and selectivity, while interpretable models provide insight into the underlying reaction mechanisms.^239^ Second, by learning from large experimental and computational datasets, ML models can accurately predict key catalyst properties, such as band gap, charge-carrier behavior, surface area, and electronic structure, facilitating rational identification of high-activity materials and heterojunctions. This creates a cycle in which mechanistic insight improves the models, and the models, in turn, accelerate the development of better catalysts. Therefore, a scalable method for property prediction and a guiding analytical framework for high-throughput research can be developed.

Descriptors are quantitative features that represent the essential physical and chemical characteristics of a system in numerical formats that machine learning (ML) models can readily process. In electrocatalysis, effective descriptors must reflect both the specific binding configurations of adsorbates and the underlying structure–property relationships that govern catalytic behavior.^240^ Effective descriptor selection should align with reaction mechanisms, ensuring that descriptors capture the key steps in nitrogen and carbon activation during electrochemical urea synthesis while avoiding strong correlations that can lead to overfitting and reduce interpretability. Once the appropriate descriptor is chosen, various machine learning algorithms, such as random forests, artificial neural networks, and support vector machines, can be employed to model and predict the behavior of electrochemical catalysts. Ultimately, the choice of algorithm should consider the dataset size, the task complexity, and the level of interpretability required for effective catalyst discovery. The research by Zhu et al. integrates DFT with ML to screen and optimize dual-atom catalysts (DACs) for electrocatalytic urea synthesis, focusing on adsorption energies, reaction barriers, electronic structure descriptors, and overall catalytic activity.^241^ The challenge in activating N_2_- and CO_2_-derived intermediates on bimetallic catalytic sites, especially in DACs, lies in the high energy barrier to N≡N bond cleavage and in competition from side reactions such as HER and nitrogen reduction reaction (NRR). Identifying effective catalysts for N_2_ activation and selective C─N coupling requires exploring a large design space, making DFT-based methods computationally expensive. To address this, the authors integrate two machine learning models with DFT. The first model uses a random forest regressor to predict a thermodynamic descriptor (E_diff_) related to overpotential, based on features such as N_2_ adsorption bond length and the d-band center. The second model predicts the N_2_ bond length based on DAC properties such as d-electron count and metal identity. SHAP analysis reveals that the d-electron count is crucial for N_2_ activation and influences catalyst performance. By combining DFT and machine learning, the authors quickly identify TaRu–Pc as a top-performing catalyst with low overpotential and high selectivity. This approach accelerates material discovery and provides valuable insights into how electronic structure affects catalytic performance in urea electrosynthesis. Zhang et al. address the complexity of studying reaction mechanisms for electrochemical urea synthesis by proposing an active learning framework that combines ML algorithms and graph theory with traditional DFT workflows.^242^ This approach efficiently explores and ranks potential reaction pathways on nitrogen-doped carbon catalysts, overcoming the computational cost of standard DFT methods. The framework uses a cascaded ML model with a gradient boosting classifier to predict the stability of candidate intermediates and a ridge regression model to estimate their formation energies. These models are iteratively applied in an active learning loop to select the most relevant configurations for further DFT analysis. This method reduces computational costs while maintaining accuracy, enabling the reconstruction of a complete reaction network with over 900 intermediates and 1,734 adsorption configurations. The framework demonstrates how machine learning can accelerate complex reaction studies and remain adaptable to other electrocatalytic systems.

## Conclusion

The PEC synthesis of urea is gaining significant attention due to its several advantages. Firstly, it utilizes abundant sunlight and readily available atmospheric pollutants such as CO_2_ and NO_3_^−^ as key reactants, with engineered catalysts to produce urea. Secondly, the process is environmentally friendly, using H_2_O as the solvent, generating minimal byproducts with no secondary pollution, and being driven by solar energy. Thirdly, it provides substantial energy savings by operating under mild conditions, eliminating the extreme heat and pressure required by conventional industrial methods. Finally, selectivity and reaction kinetics can be precisely optimized by adjusting the photoelectrode design, light irradiation geometry, and reactant selection, thereby offering enhanced control.

A key advantage of this system is the direct utilization of the photogenerated charge carriers to form the selective products, making the synthesis pathway inherently more straightforward and thermodynamically more favorable than conventional and/or advanced catalytic processes. While charge-transfer and separation mechanisms are well documented for the as-related photocatalytic, electrocatalytic, and PEC systems in the literature, this work focuses explicitly on clarifying the selective urea formation pathways at the surface. Beyond providing a general background for evaluating catalytic systems, the study summarizes recent progress by reviewing robust quantification protocols, advanced *in situ* analytical tools for tracking the urea formation yield, theoretical evidence supporting experimental findings, and demonstrating the process’s selectivity, kinetics, and thermodynamics.

The reports indicated that engineered surface defects and multiple active sites play a crucial role in enhancing reactant adsorption and facilitating C–N coupling,^120^^,^^243^^,^^244^ as discussed in detail. These features are of great importance for the initial activation of inert and abundant reactants, such as atmospheric nitrogen, at the photoelectrode surface, a substantial challenge, and a significant limitation of the field. To address this, systematic and efficient functionalization of the substrates with adjustable optoelectronic properties should be prioritized.

While PEC C–H and C–C activation is well-documented,^245^^,^^246^^,^^247^ controllable C–N activation for urea formation is still in its early stages, with a limited understanding of interfacial reaction mechanisms. Also, gathering atmospheric CO_2_ (as one of the main reactants) from the surrounding environment is not easy and demands huge costs, restricting the system’s scalability for industrial and real-world applications. Nevertheless, this emerging technology offers key advantages, including lower energy consumption, easier product separation, and high selectivity, positioning it as a promising alternative to conventional thermochemical production strategies.

Researchers have proposed an alternative approach to C–C and C–H bond activation using the combination of light and electroactive substrates, known as electrophotocatalysis, where homogenous photocatalysis integrates with electrochemical forces to drive selective reactions. Although conceptually distinct from photoelectrocatalysis, this strategy suggests practical potential for future research aimed at favorably controlling the C–N coupling trends toward higher selectivity’s. However, a key drawback is the challenge of separating the product from the homogeneous reaction environment.

The last key factor making urea photoelectrosynthesis economically viable is the development of diverse photoelectrodes suitable for large-scale applications; however, the long-term stability of PEC systems under continuous light exposure and applied voltage as operating conditions still remains unresolved, and huge efforts should be made to overcome such limitations. Overall, utilizing cost-effective raw materials and stable precursors, along with advanced surface engineering and environmentally friendly processes, can enhance the appeal of this method as a sustainable alternative to the energy-intensive conventional approaches used in the chemical industry.

### Limitations of the study

Despite the significant advances in electrocatalytic, photocatalytic, and PEC urea synthesis, several important challenges still limit their practical application and large-scale commercialization of these technologies. A major challenge is the poor selectivity of C–N coupling, as competing reactions, e.g., the HER and CO_2_-to-CO reduction, frequently decrease both urea yield and Faradaic efficiency. Also, some primary reactants of the processes, including N_2_ and CO_2_, are chemically inert due to their strong bonding energies, and this makes the activation stages on the surface challenging. Furthermore, the urea formation reaction mechanisms and C–N coupling pathways remain inadequately understood due to the limited number of potent *in situ* characterization techniques available. Catalyst stability also remains a remarkable concern, as plenty of photoelectroactive materials undergo photocorrosion, structural degradation, and loss of active sites during long-term operation. Although several studies reported promising Faradaic efficiencies, the overall urea production rates and current densities seem to be unreliable for industrial scalability, primarily because of sluggish reaction kinetics and mass-transfer limitations. Reactor engineering, therefore, remains a critical bottleneck, requiring the development of advanced flow-cell systems, gas-diffusion electrodes, and optimized membrane-based reactor architectures. Precise urea quantification is another critical challenge, since contamination from electrolytes, membranes, and nitrogen-based species may lead to overestimated results, emphasizing the necessity for standardized analytical protocols and verification approaches. Hence, future progress in this field will require standardized testing protocols, advanced catalyst engineering, deeper mechanistic investigations, reactor optimization, and long-term stability explorations to achieve efficient urea yield with the focus on sustainability and scalability.

## Acknowledgments

This work is supported by 10.13039/501100003968INSF (grant no. 40401927). Also, the authors acknowledge the partial funding from 10.13039/501100019286Ajman University under the grant no. 2025-IRG-CHS-4.

## Author contributions

M.P.: writing – original draft, supervision, writing – review and editing. E.L.: conceptualization, writing – review and editing. S.G.: visualization, writing – review and editing, methodology. A.A.: software, visualization. A.L.: investigation, writing – review and editing. A.H.-Y.: writing – review and editing. E.A.D.: writing – review and editing, funding acquisition. M.S.: writing – review and editing. L.P.: conceptualization, writing – review and editing. C.W.: conceptualization, writing – review and editing.

## Declaration of interests

The authors declare no competing interests.

## Declaration of generative AI and AI-assisted technologies in the writing process

Grammarly, a free AI writing tool, was used to check the grammar of the manuscript during the preparation steps.
